# Insight into the evolutionary and domesticated history of the most widely cultivated mushroom *Agaricus bisporus* via mitogenome sequences of 361 global strains

**DOI:** 10.1186/s12864-023-09257-w

**Published:** 2023-04-05

**Authors:** Ming-Zhe Zhang, Jian-Ping Xu, Philippe Callac, Mei-Yuan Chen, Qi Wu, Mark Wach, Gerardo Mata, Rui-Lin Zhao

**Affiliations:** 1 State Key Laboratory of Mycology, Institute of Microbiology, Chinese Academy of Sciences, No3 1St Beichen West Road, Beijing, 100101 Chaoyang District China; 2 College of Life Sciences, University of Chinese Academy of Sciences, Huairou District, Beijing, 101408 China; 3 Department of Biology, McMaster University, Hamilton, ON Canada; 4 INRAE, MycSA, CS 20032, 33882 Villenave d’Ornon, France; 5 Edible Fungi Institute of Fujian Academy of Agricultural Sciences, Fuzhou, 350014 China; 6 Sylvan BioSciences, Kittanning, PA 16201 USA; 7 Instituto de Ecología A.C. Carretera Antigua a Coatepec, 351, El Haya, 91073 Veracruz, CPXalapa Mexico

**Keywords:** Population genomics, Phylogenomic, Mitogenome, Domestication, Button mushroom

## Abstract

*Agaricus bisporus* is the most widely cultivated edible mushroom in the world with a only around three hundred years known history of cultivation. Therefore, it represents an ideal organism not only to investigate the natural evolutionary history but also the understanding on the evolution going back to the early era of domestication. In this study, we generated the mitochondrial genome sequences of 352 *A. bisporus* strains and 9 strains from 4 closely related species around the world. The population mitogenomic study revealed all *A. bisporus* strains can be divided into seven clades, and all domesticated cultivars present only in two of those clades. The molecular dating analysis showed this species origin in Europe on 4.6 Ma and we proposed the main dispersal routes. The detailed mitogenome structure studies showed that the insertion of the plasmid-derived dpo gene caused a long fragment (MIR) inversion, and the distributions of the fragments of dpo gene were strictly in correspondence with these seven clades. Our studies also showed *A. bisporus* population contains 30 intron distribution patterns (IDPs), while all cultivars contain only two IDPs, which clearly exhibit intron loss compared to the others. Either the loss occurred before or after domestication, that could suggest that the change facilitates their adaptation to the cultivated environment.

**Supplementary Information:**

The online version contains supplementary material available at 10.1186/s12864-023-09257-w.

## Introduction

Genome sequencing has provided unprecedented insights into the evolution and cultivation history of a diversity of crops and domesticated animals [1, 2]. Though many fungi have been domesticated and cultivated by humans for food for thousands of years, aside from the model yeast *Saccharomyces cerevisiae* [3, 4], little is known about the evolution and the impact of cultivation history on these fungi. *Agaricus bisporus* (J. E. Lange) Imbach, commonly called the button mushroom, belongs to Agaricaceae (Agaricales, Agaricomycetes, Basidiomycota, Fungi), which is well known by its edibility and wide distribution in the world. *A. bisporus* contains three varieties with different reproductive strategies: *A. bisporus* var. *bisporus* (J. E. Lange) Imbach 1946 [5], *A. bisporus* var. *burnettii* Kerrigan & Callac 1993 [6], and *A. bisporus* var. *eurotetrasporus* Callac & Guinb 2003 [7], corresponding to secondary homothallism, heterothallism and homothallism, respectively. This species has been cultivated since the early 1700s in France, which spread to the United States where large-scale industrial cultivation was developed, and then to other parts of the world. Presently, commercial cultivation of this species account for 61.8% of the world's total edible mushroom production and worth an estimated US $28.5 billion in 2020 [8].

In natural environments, *A. bisporus* is widely distributed throughout the world. It has been found in a diversity of ecological niches. The most common habitats are in forests of warm temperate zone with a humid climate where the ground is covered with leaf litters and the soil is rich in humus substrate [9, 10]. In western France, *A. bisporus* is among the dominant macrofungi in the litter of *Cupressus macrocarpa*, a conifer tree species introduced into this region about 170 years ago. However, *A. bisporus* has also been found in semiarid areas, including in sandy dunes without trees (French Atlantic coast) and under *mesquites* (Sonoran Desert of California). Furthermore, it’s commonly found in environments significantly associated with humans (e.g., gardens and lawns) and other animals (e.g., horse or sheep manure) [11].

The mitochondrion is a key organelle in eukaryotic cells that generate the universal cellular energy currency, adenosine triphosphate (ATP). Given the critical role of mitochondria in cellular respiration, mitochondrial DNA (mtDNA) is highly conserved among eukaryotes in its gene content. However, compared to the nuclear genome, the mtDNA is generally known to have a relatively high mutation rate, at least in animals [12]. A high mutation rate in the mitochondrial genome would enable greater discrimination among strains and populations using mtDNA markers. In addition, the high copy number of mitochondria within a single cell facilitates the analyses of their genomes using small quantities of tissue samples. Similarly, the relatively few genes in mitochondrial genomes makes it easy to identify homologous regions among evolutionary divergent organisms. Furthermore, most mitochondria are uniparentally (often maternally) inherited and are thus not recombining. These features have led to the widespread use of mitogenome sequences in species identification, phylogeography, population genetics and other fields of molecular evolution research [13–15].

Previous studies have shown that the mitochondrial genomes of fungi are significantly different from animals in mitochondrial genome size, gene number, and gene order [16, 17]. For example, in the whole-genome sequenced fungi, the mitochondrial genome sizes range from 12—272 Kb [18, 19]. Mitogenome size variations have been found not only between species but also among strains within species. Indeed, intra-specific mitogenome variations have been analyzed to study the evolutionary processes in a variety of fungi such as *Cordyceps militaris* [20], *Schizosaccharomyces pombe* [21], *Isaria cicadae* [22], *Colletotrichum lindemuthianum* [23], *Cryptococcus neoformans* [24], and *Agrocybe aegerita* [25]. These studies provided new insights into population history, gene flow, recombination, intron dynamics, and the identification of species- and population-specific genetic markers for diagnostic purposes [17, 20].

The first and currently only mitochondrial genome sequence of *A. bisporus* was published in 2013. At that time, its 135 kb mitogenome was the largest published fungal mitochondrial genome [26]. Its large size was mainly due to the presence of mobile genetic elements, including 43 group I introns and 3 group II introns for a total of 61,092 base pairs (45.3% of the entire mitogenome). In this *A. bisporus* mitogenome, the *cox1* gene was the longest mitochondrial gene (29902nt) and the largest intron reservoir including 18 group I and one group II introns. Interesting, sequence analyses showed that many of the homing endonuclease genes (*heg*) within introns had undergone degeneration [27]. A study on mitochondrial variability among 10 strains of *A. bisporus* var. *bisporus*, *A. bisporus* var. *eurotetrasporus* and a closely related species *A. devoniensis*, showed that the intraspecific evolution of the mitogenome of *A. bisporus* is characterized by a high mobility (presence/absence) of large group I introns and a low nucleotide substitution rate. The finding of low mitogenome sequence variation in *A. bisporus* has been similarly found in many other fungi, a result different from those in animals [17, 28, 29].

The mitochondrial population structure of *A. bisporus* have been analyzed in several previous studies used RFLP to analyze 441 isolates from North America and Eurasia by Xu et al. [30]. Their analyses revealed most of the 140 mtDNA haplotypes belonging to four large groups, namely the French population, the Alberta population, the coastal California population, and the desert California population. Since then, additional samples were collected, including previously under-sampled geographic regions. Notably, Callac et al. collected additional specimens from North America and Europe, and based on their life cycles and geographical distributions, divided *A. bisporus* into six populations, including two new ones: one around the Mediterranean area and the second around Europe [11]. Similarly, new wild populations were discovered in Tibet and Qilian Mountains of China [31, 32]. The whole-genome resequencing of 29 strains revealed that the Tibetan Plateau population was significantly differentiated from the European and American populations [33]. The commercial cultivars of *A. bisporus* have been studied used nuclear SSR and SNP markers, and the results showed they belonged to two groups [34, 35]. However, the patterns of intraspecific evolution and population genetic history of *A. bisporus* remain incompletely understood.

Domestication is a process of continuous selection of specific species and populations for certain traits by humans. In the early stages of domestication, natural populations with superior phenotypic characteristics were often selected as the starting materials, and after a long period of selective breeding, the domesticated populations often gave rise to many lineages with different phenotypic characteristics. Humans have domesticated important cash crops and livestock for thousands or even tens of thousands of years [1, 2, 36], and these species (e.g., rice and dogs) have now evolved many varieties that meet different human needs. As these species have been subjected to a long period of domestication, significant phenotypic and genetic differences frequently develop between domesticated and wild populations. However, their intermediate states during domestication are often not available for analyses. With advancements in whole-genome sequencing, sequence comparisons between wild strains and cultivars as well as among cultivars can provide powerful information from which to infer their domestication history. Among fungi, the origin of domestication of the Baker’s yeast *Saccharomyces cerevisiae* for fermentation and brewing and the genetic alterations following domestication have been reported [3]. However, among macrofungi, although many species (such as shiitake, *Lentinula edodes*, and *Ganoderma* spp. in China, and the button mushroom in Europe) have been consumed for a long time, there has been no analyses of the signatures of domestication on their genomes.

Among the domesticated mushrooms, *A. bisporus* is arguably economically the most important. However, its recorded domestication history was only about 300 years, much shorter than many major crops [1]. Furthermore, different from most major crops, over the past 40 years, there has been limited breeding of *A. bisporus* [37]. Indeed, the current major commercial cultivar was the first hybrid line Horst U1 developed in 1980 [38]. However, many genetic variants from this hybrid and the other hybrid U3 have been developed as cultivars, selected through either somatic or meiotic recombination and mutations. Genetic analyses of these essentially derived lines could reveal the history of cultivation and artificial selection over the last 40 years. Based on RFLP analyses, previous genetic studies have shown that two mtDNA haplotypes predominated among cultivar strains for at least the last 35 years [9, 30, 39–41]. How the mitochondrial genomes may have changed during the selection of the cultivars are not known.

In this study, we re-sequenced the genomes of 352 strains representing wild strains from all known geographic distributions and the key commercial cultivars of *A. bisporus* in the world. For comparative purposes, we also sequenced nine representative strains from four outgroup species. We are interested in the mitogenome variation and how geographic and ecological factors have likely impacted the observed genetic variations. In addition, we aim to utilize the data to infer its evolutionary history, including its origin and divergence time, as well as its potential migration and dispersal patterns, based on its current geographical distribution. Furthermore, we aim to compare the mitogenomes of domesticated and wild populations in order to investigate the differences between them and explore the potential relationship between these differences and the domestication process. This research has the potential to help the development of new cultivars of A. bisporus, or perhaps even the domestication of other edible fungi.

## Materials and methods

### Strain collection

This study analyzed a total of 361 strains, included 331 wild-collected *A. bisporus* isolates (including 284 *A. bisporus* var. *bisporus*, 45 *A. bisporus* var. *burnettii*, and two *A. bisporus* var. *eurotetrasporus*), 21 commercially cultivars of *A. bisporus*, and nine represent four phylogenetically closely related species (three from *A. qilianensis*, four from *A. sinotetrasporus*, and one each from *A.* cf*. subfloccosus* and *A. sinodeliciosus* [31]) (Supplementary Table S1). The wild *A. bisporus* strains came from all known distribution ranges of *A. bisporus* in North America, Europe, Middle East, and the new collections from Qilian National Natural Reserve and Tibet in China. Similarly, the cultivar strains were from across the globe where *A. bisporus* are cultivated.

### DNA Extraction and resequencing

To extract the genomic DNA from each strain, its mycelia were carefully scraped off the PDA medium after cultivated for one week. The genomic DNAs of all strains were extracted following the method described in [42, 43] with a minor modification. Specifically, the mycelia were rapidly frozen using liquid nitrogen and then grounded with steel balls in a homogenizer before the genomic DNA was extracted using the CTAB protocol [43]. Library construction and whole genome sequencing of the samples were performed by Biomarker Technologies Corporation (Beijing, China). The quality-checked libraries were sequenced using the HISEQ xTEN sequencing platform, and paired reads of 2 × 150 bp were obtained at approximately 100-fold coverage per strain.

### Mitochondrial genome assembly and annotation

The mitogenomes of the sequenced strains were assembled by NOVOPlasty. *Agaricus bisporus* strain H97 mitogenome (JX271275) was used as the reference and seed to bait the mitogenome sequences from other strains. The K-mer value was set to an odd number between 23 and 39. If the mitogenomes failed to auto-assemble into circular configurations, we used manual concatenation among contigs through identification and utilization of overlapping regions that are present in auto-assembled circular mitogenomes. Mitogenomes were annotated using MFANNOT and Genseq. The annotations were verified with BLAST searches of features in the reference sequence. Finally, the intron/exon boundaries were manually adjusted based on those of the reference genome JX271275.

### Phylogenomic analysis of A. bisporus mitogenomes

#### *Sequence data preprocessing and variant calling*

The raw data obtained by sequencing was filtered using Trimmomatic to remove adaptor and low-quality reads to obtain clean reads. Clean sequence reads were mapped to the *A. bisporus* mitochondrial reference genome (JX271275) using the BWA-MEM version 0.7.17-r1188 [44]. Reads were then sorted and PCR duplicates removed, using PICARD (version 2.25.6) [45]. Then, variants were determined using samtools and bcftools. Last, Snpeff verion 5.0e [46] was used to annotate and predict the effects of genetic variants on genes and proteins. In order to exclude the effect of horizontal transfer of introns, we prepared two datasets, one filtered as above and the other with SNPs in introns removed. Program circos-0.69 [47] was used to show the positions of SNPs on the reference genome for both datasets.

#### Phylogenomic analysis

Phylogenomic analysis used SNPs in the mitogenomes. SNP data were filtered for minimum allele frequency (maf) and linkage disequilibrium (LD) using vcftools (version 0.1.16) [48] and plink version 1.90b6.20 [49], and the filtered SNP data were converted to fasta format using vcf2phylip (version 2.4) [50]. Maximum-likelihood phylogenomic tree was built by RAxML version 8.2.12 [51] and *A. qilianensis*, *A. sinotetrasporus*, *A.* cf*. subfloccosus* and *A. sinodeliciousus* were used as outgroup taxa.

### Population structure analysis, and establishment of divergence times used mitochondrial genome

To determine the optimal number of genetic clusters in our sample, we analyzed allelic data at all SNP sites using a block relaxation algorithm implemented in the ADMIXTURE software [52]. The number and type of haplotypes within each genetic cluster (i.e., clade) were calculated using DNASP based on the phylogenomic tree and the population structure, and using PopART [53] to construct the haplotype network. Principal component analysis was carried out using the glPCA function of R package and using Matlab for graphing. In addition, to exclude the influence of outgroup selection on the inference of the population history of *A. bisporus*, we removed outgroup strains and used only all *A. bisporus* strains to infer their population history using DIYABC Random Forest [54]. Finally, three representative strains (with maximum genetic distance based on the position of the strian in the phylogenomic tree) from each clade were selected for divergence times estimation using the SANPP package in the beast2 software [55].

### Mitochondrial genome structure evolution at the inter- and intra-specific levels

After the mitochondrial genomes are assembled and annotated for each strain, their gene orders and intron positions and types were analyzed at both within *A. bisporus* and above the species level. For genome structural variation analysis, the mitogenomes of each clade were first analyzed for covariance using mauve to derive the approximate regions of genome structural variation. The manually calibrated annotation results were then converted to standard Genbank file format using mf2sqn per sample, then gene alignment information was exported using Phylosuit [56], and finally mapped to each sample in the evolutionary tree using Itol [57]. For intron distribution analyses, the intron annotation results of all samples were cross-checked against the reference genome JX271275. The type and structure of newly discovered introns were determined using RNAweasel [58], and their similar sequences were searched in GenBank using Blastn [59]. The relationships among introns at homologous sites in the mitogenomes were derived based on their sequence differences.

## Results

### Overview of A. bisporus mitogenomes

**Fig. 1:**
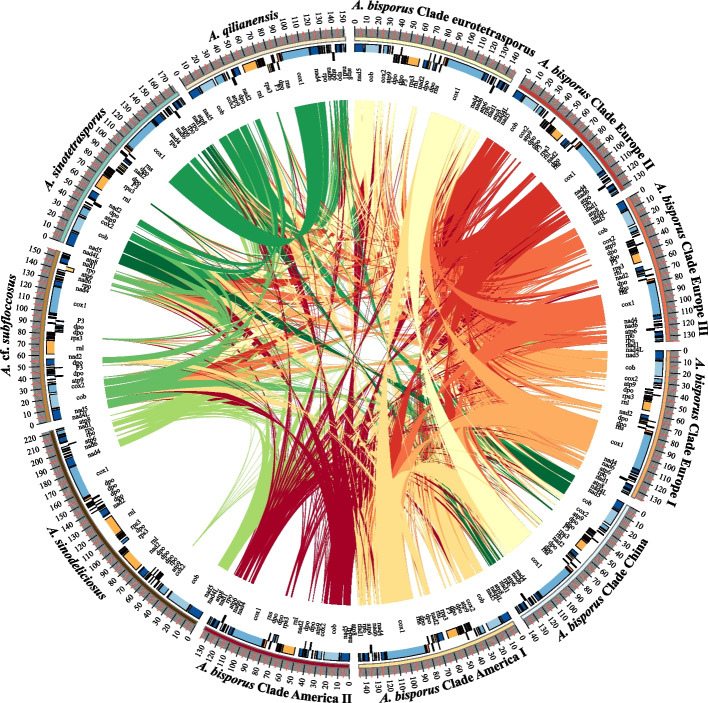
Mitogenomes of four outgroup species and seven clades of *A. bisporus*. A representative strain for each of the four outgroup species and seven clades of *A. bisporus* are selected for display (*A. sinodilicious*: ZRL20160001, *A.* cf. *subfloccosus*: BS716, *A. qilianensis*: QL20170217, *A. sinotetrasporus*: QL20170258, Clade eurotetrasporus: BS423, Clade China: ZRL20170159, Clade America I: BS305-05, Clade America II: BS109, Clade European I: BS226, Clade European II: BS134, Clade European III: BS175). The mitogenome size, and annotation information are shown in this figure. Homologous regions between genomes are connected by lines

We sequenced a total of 361 strains, and the average coverage of the mitochondrial genomes for all strains was 2224-fold. The high sequencing depth allowed us to assemble the complete mitogenome sequences for all strains. Among the five species, their genome sizes varied greatly: *A. sinodeliciosus* had the largest mitogenome at ~ 222 Kb. This was followed by the *A. sinotetrasporus* mitogenome at ~ 182 Kb. The mitogenomes of both *A.* cf*. subfloccosus* and *A. qilianensis* were about 155 Kb. Within *A. bisporus*, their mitogenome sizes varied from 134 to 148 Kb (Fig. 1 and Table 1).

**Table 1:** Genome statistics for outgroup species and seven clades of *Agaricus bisporus*

Species and Clade name	Variety and Number of samples	Location and Number of samples	Mitogenome size (Kb)	AT content	GC skew
*A. sinodeliciosus*	*-*	China (1)	222.34 ± 0.00	70.90 ± 0.00	0.026 ± 0.00
*A.* cf.* subfloccosus*	*-*	USA (1)	155.51 ± 0.00	70.60 ± 0.00	0.032 ± 0.00
*A. sinotetrasporus*	*-*	China (4)	182.11 ± 1.84	71.18 ± 0.00	0.038 ± 0.01
*A. qilianensis*	*-*	China (3)	155.76 ± 4.14	70.93 ± 0.00	0.044 ± 0.00
Eurotetrasporus	*eurotetrasporus* (2)	France (1), Greece (1)	147.40 ± 0.00	70.90 ± 0.00	0.010 ± 0.00
China	*bisporus* (6)	China (6)	138.87 ± 1.76	70.88 ± 0.07	0.014 ± 0.00
America I	*burnettii* (22)	USA (22)	148.96 ± 2.76	70.61 ± 0.07	0.008 ± 0.00
America II	*burnettii* (18) + *bisporus* (17)	USA (35)	141.29 ± 5.36	70.85 ± 0.21	0.037 ± 0.01
Europe I	*bisporus* (86)	Canada (1), France (63), Greece (19), Israel (2), Russia (1), UK (1)	137.86 ± 3.61	70.81 ± 0.08	0.012 ± 0.00
Europe II	*bisporus* (57)	cultivar (13), Canada (1), China (1), France (10), Greece (7), Mexico (3), Russia (2), UK (3), USA (17)	134.29 ± 1.25	70.81 ± 0.11	0.011 ± 0.00
Europe III	*bisporus* (144)	cultivar (9), Canada (3), France (69), France (40), Hungary (1), Russia (1), Spain (1), UK (2), USA (18)	135.66 ± 1.78	70.97 ± 0.11	0.009 ± 0.00

There was little difference in AT content in mitogenomes among the five species. Among the four outgroup species, the AT contents of *A. qilianensis*, *A. sinotetrasporus, A.* cf. *subfloccosus,* and *A. sinodeliciosus* were 70.93%, 71.18%, 70.60%, and 70.90%, respectively. Within *A. bisporus,* the mitogenome AT content ranged from 70.61% to 70.97%, with an average value of 70.87% (Table 1). Among the five species, the mean GC-skew values of *A. bisporus* (0.014) were much lower than those of the four outgroup species (*A. sinodeliciosus* 0.026, *A.* cf*. subfloccosus* 0.032, *A. qilianensis* 0.044, *A. sinotetrasporus* 0.038) (Table 1).

Fifteen protein-coding genes (*atp6*, *atp8*, *atp9; cox1*, *cox2*, *cox3*, *cob*, *nad1*, *nad2*, *nad3*, *nad4*, *nad4L*, nad5, *nad6*, *rps3*), two ribosomal protein genes (*rrnL*, *rrnS*), twenty tRNA genes (*Ala*, *Cys*, *Asp*, *Glu*, *Phe*, *Gly*, *His*, *Ile*, *Lys*, *Leu*, *Met*, *Asn*, *Pro*, *Gln*, *Arg*, *Ser*, *Thr*, *Val*, *Trp*, *Tyr*) and two polymerase genes (*dpo* and *rpo*) were discovered from all 361 mitochondrial genomes. (Supplementary Fig. 1).

### The SNP Variations of mitogenomes and phylogenomic analysis

The clean reads were mapping to the reference genome JX271275 and after filtering, a total of 7,490 SNP sites were identified among the 361 mitogenomes. These SNP sites are very robust. The highest sequencing depth of SNP sites was 2961, the lowest sequencing depth was 462, and the average depth was 1338. The lowest sequencing quality was 220, the highest sequencing quality was 228, and the average sequencing quality was 226. Only 9.24% of the 7,490 SNP sites were in known gene regions, with 4.79% in exons and 4.45% in introns. The remaining 90.76% SNPs were located in intergenic regions. Among all SNPs, 0.15% had high impact (stop gained or frameshift variant) on known genes and 99.85% had low to moderate impact (synonymous, missense, and non-coding variant).

**Fig. 2:**
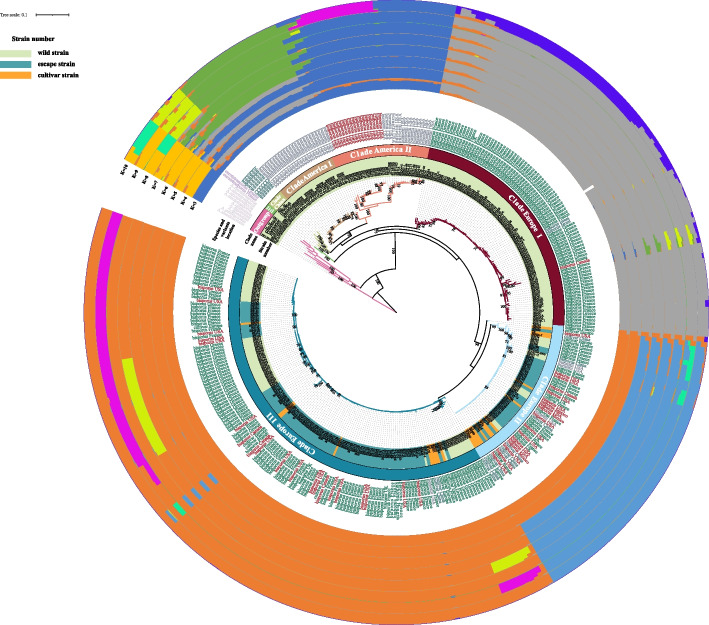
Phylogenomic and population structure analysis. 1666 SNP data from the mitochondrial genomes of 361 strains were used. The Outgroup and the seven clades in which *A. bisporus* are divided are marked in the figure. The genuine wild strains, cultivar-like strains and cultivar strains are distinguished by a different color on the strain name. The outer circles show the results of the population structure analysis for K values of 3 to 9, respectively

**Fig. 3:**
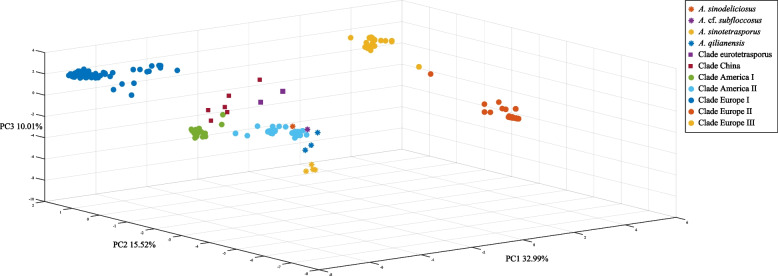
Principal component analysis. The same SNP data as for Fig. 2 were used for principal component analysis and a 3-dimensional stereogram was constructed using the first three principal components. The four outgroup species and the seven clades of *A. bisporus* strains are represented using different shapes and colors

Phylogenomic trees were built based on two filtered datasets, one including all SNPs and the second without SNPs from introns (SNPs information see Supplementary Fig. 2), and both used *A. sinodeliciosus*, *A.* cf. *subfloccosus*, *A. sinotetrasporus* and *A. qilianensis* as outgroups. Both phylogenomic trees showed the same topology, and the tree based on all SNPs was shown in Fig. 2 and Supplementary Fig. 3. Seven different clades of *A. bisporus* were inferred: 1) Clade Eurotetrasporus, 2) Clade China, 3) Clade America I, 4) Clade America II, 5) Clade Europe I, 6) Clade Europe II, and 7) Clade Europe III. This is consistent with the result of PCA (Fig. 3). The strains information in each clade is shown in Table 1. The basal clade contained the two strains of *A. bisporus* var. *eurotetrasporus*, which we named it Clade Eurotetrasporus. The strains in Clade America I, II, and China all come from a single geographic region, so they are named by geographic source. Clade Europe I contains strains from Europe, except for one from Canada and one from Israel. The remaining two clades contained strains with mixed geographic origins, including all 22 cultivar strains and 178 “wild” strains collected from Europe and North America. Because most wild strains in these two clades were from Europe, we named these two clades Europe II and Europe III.

### Population structure of A. bisporus and their divergence times

In the population structure analysis, we observed a gradual stabilization of the CV values as K values increased to 6 (Supplementary Fig. 4), and the increase in K values (from 6 to 9) did not allow new groups to be delineated (Fig. 2). This situation has been reported several times in studies of other species [60–62]. This result is consistent with the results of phylogenomic analyses, except that two distantly separated group, Clade Eurotetrasporus and China, show similar and diverse compositions at the same time.

Based on the divided clade, we calculated the genome size, AT content and GC skew of each clade of *A. bisporus* (Table 1). The values were consistent among strains within each clade and differed significantly between clades. Notably, the size of the mitogenome was related to their clustering patterns on the phylogenomic tree, as shown by the size of the basal branch (Clade Eurotetrasporus) and the first branch (Clade Europe I, China, America I, and II) being larger than the other branch (Clades Europe II and III). In contrast, the average GC skew of Clade America II was more similar to that of outgroup species than to its closest clade.

**Fig. 4:**
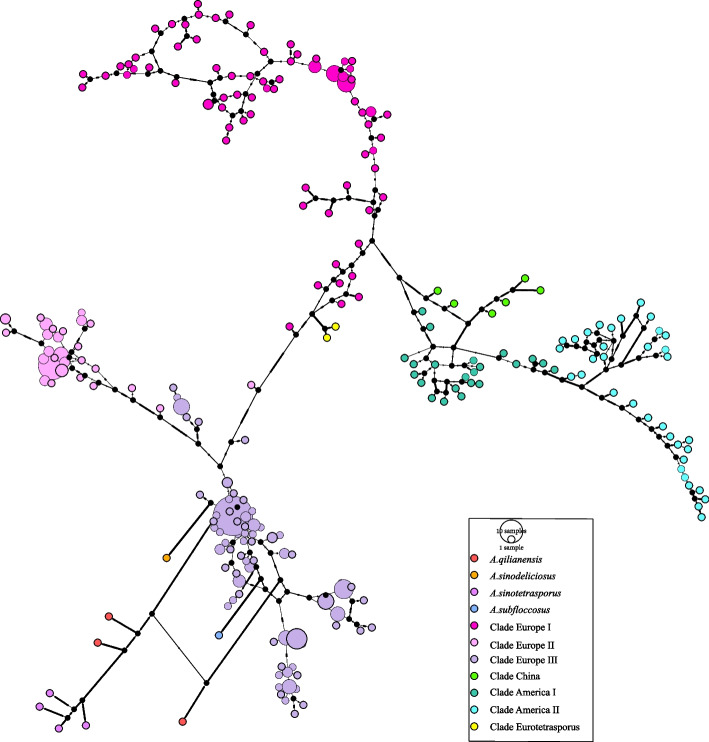
Haplotype network structure analysis. The same SNP data as for Fig. 2 were used for haplotype network structure analysis. The four outgroup species and the seven clades of *A. bisporus* strains are represented using different colors of Circles. Each circle represents a haplotype, with larger diameter circles representing more strains with this haplotype

A total of 245 haplotypes were found among the 361 strains (Fig. 4). The Clade Eurotetrasporus is located closest to the center of the diversities, and all the remaining samples diversified from them. There were two major evolutionary directions: one diverged into the Clades Europe II and III, and another into the remaining four clades, which were Clades Europe I, China, and America I and II. The results of network structure analysis also supported the conclusion that Clade Eurotetrasporus was closest to the ancestral population of *A. bisporus*. However, we note that even though the diversification among the mitogenome clades seemed clonal, we found evidence of loop structures within individual clades (Clade Europe I), consistent with recombination in the mitogenomes of this species.

In order to infere and test the history of the *A. bisporus* population, we predicted three possible scenarios (Supplementary Fig. 5), which were evaluated using the DIYABC-RF software. The results show that the scenarios 1 have the highest probability, which is consistent with our Phylogenomic trees (Supplementary Fig. 6).

**Fig. 5:**
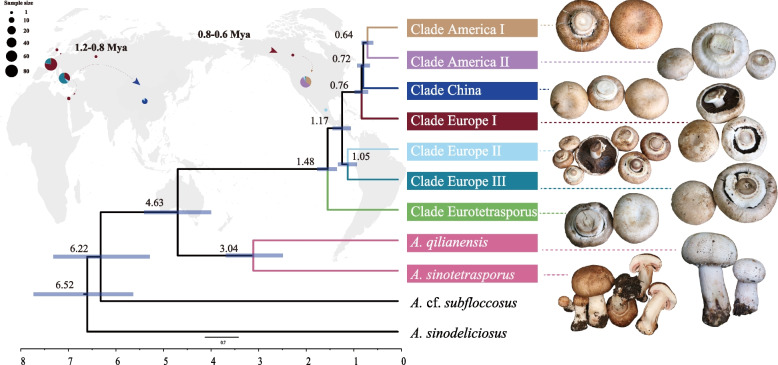
Inference of divergence time, transmission routes. The diagram on the left shows the inferred divergence times and migration events of *A. bisporus*. The divergence times of each branch are indicated. The diagram on the right shows the presumed route of its spread

SNAPP was used to estimate the divergence times of each clade. Our dating analyses suggested that *A. bisporus* likely diverged from *A. qilianensis* and *A. sinotetrasporus* around 4.62 million years ago (Ma). Within *A. bisporus,* Clade Eurotetrasporus, originated around 1.47 Ma; Clades Europe I split from II and III around 1.17 Ma; the ancestor of Clade Europe I dispersed from Europe around 0.76 Ma, then formed the Clades China and America I and II respectively around 0.72 Ma (Fig. 5). The oldest diverged branch Clade Eurotetrasporus contains the only two strains of the *A. bisporus* var. *eurotetrasporus*, which are characterised by homothallism reproduction, while the heterothallic strains in *A. bisporus* var. *burnettii* were found in the youngest Clades America I and II. All strains in other clades belonged to the the secondary homothallic *A. bisporus* var. *bisporus*. The above analysis suggest that the mode of reproduction in *A. bisporus* likely started as homothallic from which secondary homothallism and then heterothallism evolved.

### Mitochondrial genome evolution at the inter- and intra- species levels

#### The order of genes between two inverted repeats sequences (IRS)

**Fig. 6:**
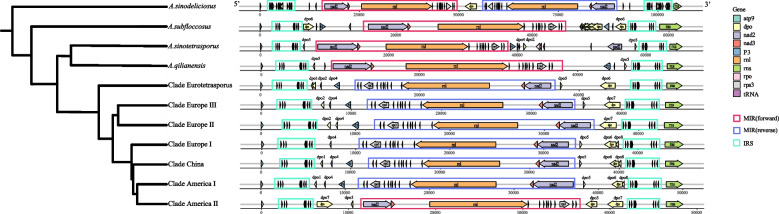
Structural differences in the mitogenomes of the four outgroup species and the seven clades of *A. bisporus*. The mitogenomes of seven clades of *A. bisporus* and four outgroup species have distinct structural differences in the region between atp9 and rrnS. Each genome has two segments with the same sequence and in opposite directions, named IRS. A segment between IRS has different direction in different clades, named MIR

All strains contained a pair of inverted repeats sequences (IRS) consisting of eight tRNA genes within each repeat. In *A. sinodeliciosus,* the IRS consists of both tRNA and *dpo* gene fragments. Between those two IRSs there is a sequence which we call MIR, short for the Middle of two Inverted Repeats, consisting of 9 *tRNA*, *nad2*, *nad3*, *rrnL*, *rps3*, and several *dpo* gene fragments, which may differ among the clades in the directions of transcription (forward or reverse order) (Fig. 6).

With the exception of *A. sinodeliciosus* and a very small number of *A. bisporus* strains (3 strains, one each from Clades Europe I, II, and III) which have repeated MIRs in two different directions, all remaining strains have only one MIR sequence. The MIR sequences for all strains in *A.* cf*. subfloccosus*, *A. sinotetrasporus*, *A. qilianensis* are in a forward direction. However, most strains of *A. bisporus* (317/352) contain MIR sequences in reverse direction, and the remaining strains (32/352) are in a forward direction. Among all seven clades of *A. bisporus,* the vast majority of strains containing the forward MIR were located in Clade America II (27/35), with the remaining few distributed in Clade China (1/6), Europe I (1/86) and II (1/57), and Europe III (2/143) (Supplementary Fig. 1).

#### Diversity of mitochondrial gene dpo

**Fig. 7:**
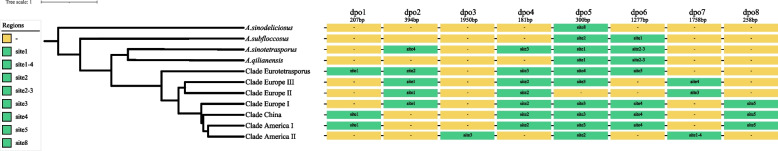
*dpo* fragments distribution in each Clades. A total of eight different fragments of the dpo gene were found in *A. bisporus*. The diagram shows the length of each dpo fragments and its distribution in each clade. Green blocks indicate the presence of the dpo fragment and, conversely, yellow blocks indicate the absence of the fragment. Site* indicates the ranking of the dpo fragment in the genome of the clade

Another variation in protein-coding genes is found in the *dpo* gene. The *dpo* gene is known as plasmid origin and codes for a DNA-directed DNA polymerase. Among our sequenced strains, a total of eight fragments of *dpo* gene were identified in *A. bisporus*,which were located midway between the IRS and MIR. Based on their positions, these fragments were named *dpo* 1–8 (Fig. 6, 7). Among strains within each clade, the number and locations of the fragments of *dpo* gene were almost identical to each other, while they were different among clades. Based on the distributions of *dpo* gene fragments in the seven clades of *A. bisporus* and their positions in the phylogenomic tree, we can find that the basal clade (Clade Eurotetrasporus) contains five fragments *dpo*1, 2, 4, 5, and 6. Clades Europe II and III contain almost the same *dpo* composition (*dpo* 2, 4, 7), plus Europe III has an additional *dpo* 5. In another branch, Clades Europe I, China and America I also have almost the same *dpo* composition (*dpo* 4, 5, 6, and 8), the difference being that Clade China and America I have an additional *dpo* 1, while Europe I has *dpo* 2. The difference is in America II, which contains *dpo* 5, *dpo* 7 and the unique dpo3.

#### Diversity of introns in A. bisporus

##### The unique introns and new intron types in A. bisporus

As mentioned before, a total of fifteen protein-coding genes have been found in the mitochondrial genome in *A. bisporus*. In seven of the 15 genes (*nad5*, *cob*, *cox2*, *cox1*, *nad4*, *nad1*, and *cox3*), 55 group I introns and 5 group II introns insertion sites were found among 352 strains of *A. bisporus* and 9 strains of four outgroup species. Presence or absence of introns at these 60 sites in each strains are recorded in Supplementary Table S2. In this Table, the 60 introns are numbered following the order of their insertion sites in each gene and the subclasses of introns are indicated. Of these 60 introns, 13 are not variable and present in all samples of all species. In contrast, in 9 of the 26 insertion sites located in *cox1* gene, we observed three instances of variability other than presence/absence. In the first, two different types of introns were found at the same insertion site (sites 12, 15 and 26), and three different types of introns were found at site 23. Previous research reported only the two types of intron at site 12 iAbi9 and iAbi9’ [27, 28] (Supplementary Fig. 7). In the second, intron degeneration was found in* A*. cf. *subfloccosus*, where partial sequences of intron 3 and intron 4 were deleted (Supplementary Fig. 7). Third, insertion sequences were found within three introns: a 18 bp insertion was found in intron 5 of all samples of *A. qiliensis* and *A. sinotetrasporus* but not in other strains analyzed here. Similarly, a 1238 bp sequence was inserted in intron 13 of some strains of *A. bisporus,* likely originated from a *cob* intron according to [27]; and a 1162 bp sequence was inserted in intron 18 in samples of *A*. cf. *subfloccosus*, *A. sinotetrasporus* and some samples of *A. qilianensis.* This insert sequence is highly similar to *cox1* introns of *Tuber calosporum* (73% of identities) and *Dactylella tenuis* (78% of identities).

We note that intron 25, intron 15 type 1, intron 26 type 2, and insert sequence within intron 13 were only found in *cox1* of *A. bisporus* but not found in the nine strains of outgroup species. In addition, intron 26 type 2 is species specific since it is present in all samples of *A. bisporus,* whereas all samples of other species of *Agaricus* have the intron 26 type 1*.*

##### The introns distribution pattern (IDP) in A. bisporus

**Fig. 8:**
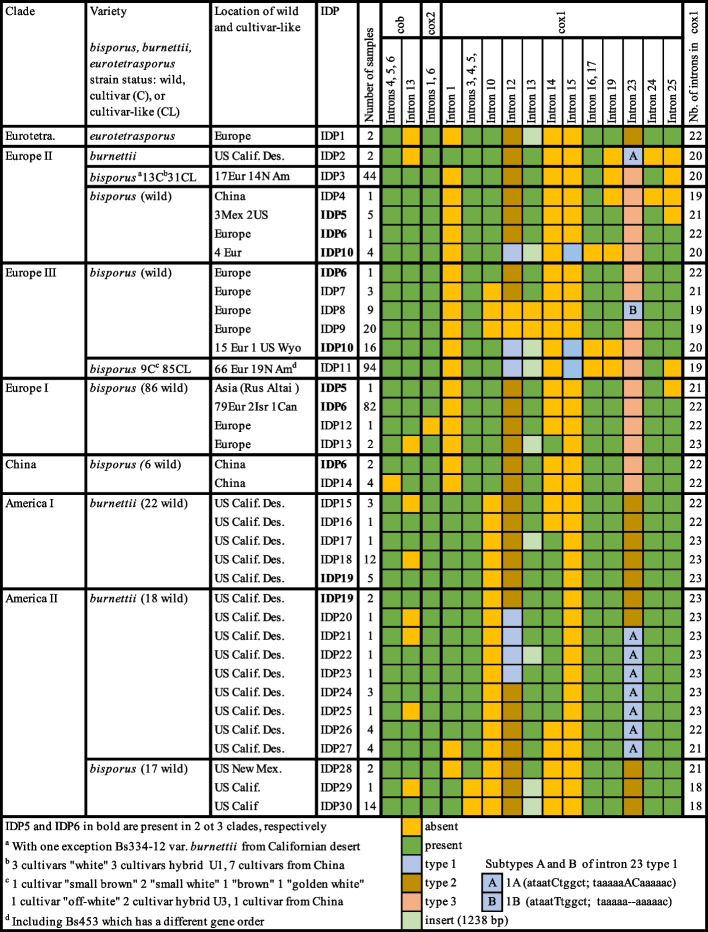
IDP in each Clades of *A. bisporus*. A total of 30 different IDPs (intron distribution patterns) were found in *A. bisporus*. The diagram shows introns that differed among the clades and identical introns were removed. The diagram shows the IDPs for each clade, the origin of the strains and its number in each IDP, and the number of introns in cox1

In *A. bisporus,* introns are found at 44 of the 60 intronic sites (41 group I and 3 group II introns). At 22 of these 44 sites, the same intron is present in all strains of *A. bisporus*. The remaining 22 variable sites are all located in three genes *cob, cox2* and *cox1*. In Supplementary Table S2 the strains were first arranged in the same order as in the phylogenomic tree based on SNPs. Since samples in the same clade and/or closely related to each other often had the same introns, they were easily classified according their intron distribution pattern (IDP). Samples of *A. bisporus* belonging to the same IDP class have exactly the same introns at all 22 variable intronic sites. We have listed 30 different IDP classes in Table S2 and 30 of them, which are found in *A. bisporus* and only in this species are listed in the simplified Fig. 8. Information included in this Table reveals congruence between these 30 IDP classes, the SNP-based phylogeny, the taxonomy at the varietal rank, and the geographical or farm origins of the strains.

The IDPs from different clades are greatly distinguished from each other (Fig. 8). For example, 26 of the 30 IDP classes are found in only one of these clades. However there are also some IDPs which are shared within 2 clades, such as Clade Europe I and II share IDP5; Clade Europe II and III share IDP10; Clade America I and II share IDP19. The most popular IDP is IDP6 which is found in four clades (Clade China, Europe I, II and III), and that form a group paraphyletic to the group composed of Clade America I and II. This suggests that the history of introns has broadly followed the evolutionary history of the mitochondria and that IDP6 could be an ancestral Eurasiatic IDP.

IDP classes are also deeply correlated with taxonomy. Here, samples of *A. bisporus* var. *eurotetrasporus* are in the single class IDP1 and samples of *A. bisporus* var. *burnettii* are in 14 classes, which are IDP2 in clade Europe II and classes from IDP15 to IDP27 in clade America I and II. Samples of *A. bisporus* var. *bisporus* are in the remaining 15 IDP classes. A unique exception is strain Bs334-12 which is the only sample of *A. bisporus* var. *burnettii* among the 44 samples with IDP3. The results are consistent with biological or geographical isolations of the three varieties. It is remarkable that 47% of the IDP classes (14/30) are found in *A. bisporus* var. *burnettii*, although this variety is much less represented in this study than *A. bisporus* var. *bisporus* and with all 14 strains from the same geographic region, namely the Sonoran Desert of California.

As described above, IDP classes are largely correlated with their geographic distributions. However, there are two notable exceptions. Our wild North American samples were from Canada, Mexico and USA, and our Eurasian samples were from Europe and Asia (Altai Republic of Russia, China and Israel). Among 30 IDP classes, 17 contained samples exclusively from North America and 11 exclusively from Eurasia with the exception of three samples: one sample of IDP5 was from Russia, one of the 82 samples of IDP6 class was from Canada, and one of the 20 samples of IDP10 class was from USA. Only in the two remaining classes IDP3 and IDP11 are wild samples from the two continent, North America and Europe, significantly represented. In addition, all 21 cultivars studied belong to these two IDP classes. Wild samples with IDP3 and IDP11 are traditionally called ‘cultivar-like’ because they have the same mitochondrial genotypes as all known cultivars based on previous RFLP genotyping analyses. Here, the class IDP3 includes 31 cultivar-like samples (17 from Europe and 14 from North America) and 13 cultivars including the traditional “white” type, the hybrid “U1” type and strains cultivated in China. The class IDP11 includes 85 cultivar-like (66 from Europe and 19 from North America) and 9 cultivars including traditional “off white”, “golden white”, small white”, “brown”, and “small brown” types, the hybrid “U3″ type and a strain cultivated in China.

##### Notable introns showing presence/absence polymorphism

Interestingly, there are four cases showing evidence of probable simultaneous loss of several neighboring introns. First, introns 4, 5 and 6 within gene *cob* are missing only in the four samples from China and they constitute the class IDP15. Second, introns 1 and 6 of *cox2*, which are neighboring in *A. bisporus*, are both missing in one sample from France and represents the IDP13 class. Third, introns 3, 4, 5 and 6 of *cox1* are missing in the 15 samples from California and they constitute the classes IDP30 and IDP31. Since these two classes are highly similar to each other, the simultaneous loss of these four introns likely occurred in a common ancestor of these 15 samples. Fourth, introns 16 and 17 of *cox1* are missing in all 114 samples of the highly similar class IDP10 and class IDP11 (comprising cultivars) and thus might have been lost in their common ancestor. Curiously, the neighboring intron 15 (intron type 1) is only present in IDP classes 10 and 11. Together, the losses of these 11 introns (4, 5, 6 of *cob*, 1, 6 of *cox 2*, 3, 4, 5,6, 16, 17 of *cox1*) could be resulted from as few as four events.

The remaining 10 introns showed more complex distributions. Here, we will focus on the distributions of introns 1 and 23 of *cox1*. Intron 1 is present only in samples from America, more precisely in 51 of 57 samples of clades America I and II (all 16 IDPs except IDP 27 and IDP 28) and in two samples of var. *burnettii* from America (IDP 2) which are unexpectedly placed in clade Europe II. It is of course absent in classes IDP 3 and IDP 11 which contain cultivars and 33 cultivar-like strains from America.

The intronic site 23 was found to contain intron in all strains analyzed here, including in *A. bisporus* and in its closely related species (Supplementary Information). Based on differences in sequences, the introns at this site can be classified into three types. Type 1 is present in classes IDP21 to 27 and class IDP2 of var. *burnettii*, which are in clade America II and clade Europe II, respectively. Type 2 is present only in clades Eurotetrasporus and America I and II. Type 3 is present only in clades China, Europe I and Europe II and III. However, intron 23 type 1 is also unexpectedly present in the 9 wild samples of var. *bisporus* of IDP8 class in clade Europe III (Fig. 8.). As this distribution was unexpected, sequences of type 1 were further examined and two polymorphisms (a SNP and a two-bp indel) were found to distinguish two subtypes 1A and 1B as indicated in Fig. 8. The samples of var. *burnettii* from California (IDP22 to 28, and IDP2) are of type 1A and the samples of var. *bisporus* from Europe (IDP8) are of type 1B.

## Discussions

### Summary of the mitochondrial genomes of A. bisporus

In this study, we obtained the mitogenome sequences of 352 strains of *A. bisporus,* including all known distribution ranges of this species in the world and representative commercial cultivars, and 9 strains of its closely related species. The size of fungal mitochondrial genomes varies greatly in different species, such as the largest published mitochondrial genome among phylum Basidiomycota is 256 Kb in *Clavaria fumosa* Pers., the largest in Fungi is 272 Kb for *Morchella importuna* in Pezizomycetes, while *Rozella allomycetis* in Cryptomycota has the smallest known mitochondrial genome (12 kb) in Fungi [18, 19, 24].

The size of mitochondrial genomes within *A. bisporus* was diverse, ranging in size from 134 to 148 Kb, which is similar to or larger than previously reported 135 kb [26]. In our study, the mitochondrial genome sizes of different clades of *A. bisporus* and outgroup species differed significantly too. For example, the differences between Clades Europe II and America I are 14 kb, and differences between Clade Europe II and *A. sinodeliciosus* are 77 kb. Almost all the differences were due to length variations in intergenic regions and introns. Our observations are consistent with those from previous studies showing limited differences in protein-coding genes among fungal mitogenomes within species, with most protein-coding genes related to oxidative phosphorylation [14].

The mitogenome AT contents among strains within each clade were similar to each other (Table 1). However, among clades, there were significant differences in AT, supporting the distinctiveness of the identified clades. GC skew is commonly used to distinguish leading and lagging strands as well as to mark the start and end points of circular DNA. We calculated the average GC skew of all mitogenomes (Table 1) and found that the average GC skews of six *A. bisporus* clades ranged from 0.008–0.013. Interestingly, Clade America II had a GC skew similar to the outgroup species, at around 0.037. We attribute this phenomenon to the similar mitogenome structure between Clade America II with the outgroup species. This hypothesis is confirmed in our following analysis about the direction of the IRS.

Our data show that all clades of *A. bisporus* and the four outgroup species contain 15 protein genes, 21 tRNA genes, 2 ribosomal protein genes and two polymerase genes. *A. bisporus* was once thought to have 43 group1 introns and 3 group2 introns, making it the largest known reservoir of group 1 introns in eukaryotes. In this study we found a total of 44 intronic sites in this species, including with three different introns types at the same intronic sites. In particular, the *cox1* gene contains the largest number of introns and their number, sequence types and combinations contributed to intraspecific mitogenome variation.

### Speculation on the evolutionary history of A. bisporus

The mitochondrial genomic SNP data were used to construct the phylogenomic tree and infer the population structure. Our phylogenomic, population genetic structure and haplotype network structure analyses indicated that *A. bisporus* strains can be divided into seven clades, named as Clades Eurotetrasporus, China, America I and II, Europe I, II, and III.

Mitochondria of European samples (Clades Eurotetrasporus and Europe I, II, III) are likely the first diverged and closest to the ancestral mitochondrial genome in *A. bisporus*. Indeed, the two strains of *A. bisporus* var. *eurotetrasporus* were isolated from Greece and the French Atlantic coast [7], and they constitute the most basal clade within the species (Clades Eurotetrasporus) with the divergence time of around 1.47 Ma (Fig. 5). In addition, it is centrally located in the haplotype network structure diagram, and contains ancestral elements linked to all other clades in *A. bisporus* (Fig. 4). On the other hand, based on the distribution information of *A. bisporus* var. *eurotetrasporus,* the population size might be quite small as it only represents less than 1% of the specimens of *A. bisporus* collected in Europe. Presently, Europe is the only known distribution of this variety. However, we cannot exclude the possibility that its geographical distribution range could be larger.

Due to its homothallic lifecycle and its close resemblance to outgroups in its mitochondrial genome, *A. bisporus* var. *eurotetrasporus* looks like the most ancestral form in this species, in comparison to the mitogenomes of other varieties. The switches in reproductive mode is a complex process and transitions between the heterothallism to homothallism are common among species within the same genus, or even among strains within the same species [7]. The switch could be induced by both environmental and genetic factors and could represent a survival strategy [63]. Among fungi, evidence for both a shift from homothallic to heterothallic and from heterothallic to homothallic have been observed [64–66]. Our findings using the mitogenome sequences suggest switches from homothallism to secondary homothallism and to heterothallism in *A. bisporus*. However, because mitochondrial genomes are not known to determine mating system, the exact genetic changes causing switches in reproductive systems among the varieties await data from the nuclear genomes.

As the nearest known relatives of *A. bisporus*, species *A. qilianensis* and *A. sinotetrasporus*, are found in the Qilian Mountains of China, we speculate that the common ancestor of these three species may have been widely distributed in Eurasian continent. However, due to the onset of the ice age with cold temperatures, only populations at high altitudes were able to survive and reproduce, and the geographical isolation of populations led to the formation of new species. It was not until 4.6 Ma that a new species, the *A. bisporus*, was formed on the European continent. Populations of *A. bisporus* began to diverge as a result of environmental influences, the earliest of which diverged at 1.47 Ma to form Clade Eurotetrasporus, then form two special traits group Clade Europe II and III at 1 Ma. After further dispersal, the group that continued to survive in Europe formed Clade Europe I at 0.76 Ma and the group that returned to China formed Clade China at 0.72 Ma, which spread via the Bering Land Bridge to the North American continent and gradually diverged to form the distinctive groups Clade America I and II (Fig. 5). Our proposed route of dispersal and divergence for *A. bisporus* has been similarly reported for other taxa [67, 68]. In this hypothetical scenario, the unique geography of the Tibetan Plateau likely played an important role in generating and maintaining the genetic diversity of *A. bisporus* (Clade China) and its closely related species (*A. sinotetrasporus* and *A.qilianensis*) [32].

### Characterization of the mitogenomes of domesticated clades of A. bisporus

By examining the genetic composition and mitogenome structure of all strains, we found two notable differences among clades of *A. bisporus.* The first is the orientation of the MIR sequence, which is consistent among all strains within each clade. However, there were differences between *A. bisporus* and outgroup species and that Clade America II of *A. bisporus* had a unique structure (Fig. 6). The second is in the *dpo* gene where there were significant differences in the type and number of *dpo* gene fragments among *A. bisporus* clades (Fig. 6, 7).

The *dpo* genes encode DNA polymerase and are related to mitochondrial plasmids. However, the fragments of *dpo* gene often insert into mitochondrial genome. A similar situation had been found in *Agrocybe aegerita* [25, 69] and in *Moniliophthora perniciosa* [70]. An early study on mitochondrial genome of *A. bisporus* also revealed the presence of four *dpo* fragments with a plasmid origin, two of which within the IR (*dpor1* and *dpor2*) and the remaining two found between the IR (*dpo1* and *dpo2*) [26].

In our study, we found a total of eight different *dop* gene fragments in the mitogenomes of the analyzed strains (named as *dpo* 1–8). Among the species, these *dpo* gene fragments differed in their sites of insertion in the mitogenomes as well as in their nucleotide sequences. Similarly, the distributions and nucleotide sequences of these eight *dpo* gene fragments were also different among the clades within *A. bisporus*. Within individual clades of *A. bisporus*, the different combinations of those eight *dpo* fragments are generally consistent, the exception was within Clade America II where variations in *dpo* fragment distributions were found among strains.

The observed pattern of the *dpo* distributions suggests that the insertion of *dpo* from the plasmid into the mitogenome of *A. bisporus* was likely rare and ancient. We therefore hypothesize that changes caused by the insertion and loss of *dpo* fragments reflect the divergence among clades of *A. bisporus.* Although we currently do not know the effects of *dpo* insertion on the mitogenome of *A. bisporus*, the very stable structural difference between the cultivated and non-cultivated clades suggested that the specific *dpo* distribution pattern or its related genetic changes which we do not known presently in the mitogenome might have enabled these strains to be more successful at being domesticated by humans. In *Neurospora* spp., insertion of plasmid DNA into the mitochondrial genome leads to the disruption of several genes, resulting in senescence and respiratory defects leading to death [71–74]. In contrast, in *Podospora anserina* and *Physarum polycephalum*, the insertion of plasmid genes into the mitochondrial genome led to increases in their lifespan [75–77]. Further studies on the function of *dpo* would be very helpful to understand their effects on the cultivation of *A. bisporus*.

These *dpo* genes are usually thought to be acquired by plasmid integration into the mitochondrial genome, but when and in what way it is integrated into the mitochondria remains to be studied. An early study of *rpo* (encode RNA polymerase), also derived from plasmids and closely related to *dpo*, suggests that a common sequence ancestor of pEM plasmids and mitochondrial RNA polymerase-like sequences may have existed prior to the formation of the *Agaricus* species [78]. The presence of the same *dpo* gene fragment that we found in *A. bisporus* as well as in four outgroup species also indicates that the initial insertion of *dpo* occurred prior to the divergence of *Agaricus* into these five different species. During the divergence and speciation both within *Agaricus* genus and within *A. bisporus*, additional insertions and/or losses of *dpo* gene occurred, leading to the current pattern.

### Mitochondrial genome evolution of A. bisporus under domestication

#### Intronic variability

Among the 44 intron insertion sites, 22 found in six mitochondrial genes are present in all 352 samples of *A. bisporus*. The remaining 22 intronic sites located in three genes are polymorphic among the strains and these introns classified all *A. bisporus* strains into 30 IDP classes (Fig. 8). The strong correlation between these IDP classes, the major phylogenomic clades based on mitogenome SNP profiles, and the varietal taxa suggest that these IDP classes are relatively stable and that loss and gain of introns were rare events. However, the high frequency IDP (14/30) in *A*. *bisporus* var. *burnettii* was surprising. Our results are consistent with a previous report by Xu et al. [30] that revealed a higher number of mitochondrial haplotypes within this heterothallic variety. A recent study revealed that sexual mating can promote the transmission of intron introgression in the basidiomycete yeast *Cryptococcus neoformans* [79]. The greater intron number in the heterothallic var. *burnettii* is consistent with what was found in *C. neoformans.*

As shown in Table S2 and Fig. 8, the average number of introns in *cox1* in the 14 IDPs of var. *burnettii* and one IDP of var. *eurotetrasporus* was 22.4, higher than that (20.3) in the 16 IDPs of var. *bisporus*. In *A. bisporus*, except for the two IDPs where only 18 introns were found in *cox1* gene (IDP29 and IDP30), the average number of introns in Clade Europe I is 22, while the average number of introns in Clades Europe II and III is 20. The results suggest that the loss of introns is particularly pronounced in clades Europe II and III, the two groups containing cultivars of *A. bisporus*.

Neighboring introns could be simultaneous lost when they are located in the same re-proprocessed segment [80]. In such a process, mRNA is reverse transcribed and integrated back into the genome without the introns. Could such a process be more frequent or occur only in var. *bisporus*? Our observation of a lower number of introns in var. *bisporus* than in other varieties and clades is consistent with a “yes” answer to this question. In other respects, there are four elements which could have been gained in *cox1* gene of *A. bisporus*. Indeed, intron 25, intron 15 type 1, intron 26 type 2 and the 1238 bp insert into intron 13 were not found in the closely related species. Intron 26 type 2 which is present in all samples of *A. bisporus* as a specific marker likely transferred into this species early. Using nBLAST, the closest sequence to the intron 26 type 2 sequence is found in *Leucoagaricus naucinus* (85% identity with a *cox1* intron in *Leucoagaricus naucinus*). On the contrary, intron 15 type 1 (88.07% identity with a *cox1* intron in *Russula abietina*) is found only in classes IDP10 and IDP11, and could have been initially transferred in a common ancestor of these two IDP classes. Intron 25 and insert of intron 13 have more complex histories that cannot be easily reconstructed. Intron 25 (77.73% identity with a *cox1* intron in *Drechslerella brochopaga*) is present in all seven clades of *A. bisporus*, but is missing in some strains of Clade Europe II and III (IDP2, 3, 4, 5, 11) and in one strain of Clade Europe I (IDP5), which appears to be a combination of an ancient gained event and recent loss events, which were also associated with domestication. Intron 13 (69.33% identity with a *cox1* intron in *Trametes cingulate*) is present in all outgroup strains, however, some strains of Clade Europe III (IDP 8 and 9) in *A. bisporus* have lost this intron. The 1238 bp insert into intron 13 is present in some strains of the remaining clades except for Clade China. Thus the insertion of intron 13 likely occurred prior to the differentiation of its population, followed by the loss of the insertion sequence and the loss of intron 13 as a whole. Overall, the high but variable sequence identities of intron sequences in *A. bisporus* with those from divergent taxa suggest continuous intron invasions during the evolutionary history of *A. bisporus*.

#### Intron distribution patterns of cultivars

All the cultivars have mitogenomes in the IDP3 and IDP11 classes of the Europe II and III clades. Furthermore, previously studies have shown that mitogenomes from cultivars have introgressed into wild population of this species [9, 30]. Indeed, samples from both North America and Eurasia are found to have mitogenome intron patterns belonging to these two classes. In contrast, strains from each of the 27 other IDP classes are exclusively from one continent (North America or Eurasia) with only 3 exceptions (IDP5, IDP 6, and 10). The results suggest that mitochondria rarely migrated naturally between continents and that mitochondria from cultivars which have IDP3 or IDP11 migrated through human activities in agreement with the history of European cultivars that have been introduced and cultivated in other continents by spawn makers and/or international mushroom trade. Using 9 RFLP markers, Xu et al. [30] found that most cultivars of *A. bisporus* belonged to two mitochondrial haplotypes named mt1 and mt2. Many wild strains having these two haplotypes also had nuclear genotypes similar to cultivars and such wild strains were called cultivar-like. In our current sample, 29 cultivar or cultivar-like strains having the mt1 haplotype and 13 having the mt2 haplotype in the study by Xu et al. [30] corresponded to the IDP11 and IDP3 classes, respectively. This indicates that polymorphisms revealed by RFLP in the Xu et al. [30] study were mainly due to presence/absence of introns and the types of intron and inserts, which were unknown at that time. These two IDPs of cultivars are in clades Europe II and III in agreement with their presumed European origin.

#### Hypothesis on the effects of domestication

With the discovery of mycelium culture and spore germination under axenic conditions, breeding began in the early twentieth century, but with the industrial production of grain spawn by a few spawn makers, the number of cultivar types offered on the market was reduced to about six in the 1970s (white, off white, small white, golden white, brown and small brown). In the early 1980s, two hybrids U1 and U3 were obtained by crossing “white” and “off white” cultivars [38, 40]. Since then, other commercial cultivars have been developed but most of them were derived from U1 and U3 [38] through selection with or without mating. Hybrid cultivar U1 inherited the mitochondria of its “white” parent cultivar and both are in the same IDP3 class. Apparently, hybrid cultivar U3 inherited the mitochondria of its “off white” parent cultivar and both are in the same IDP11 class. What is interesting is that the other traditional cultivars brown, small brown, small white and golden white are also found to have the IDP11 even though they have different phenotypes, distinct genotypes, and different domestication histories. For example, previous studies revealed that “off white” and “white” cultivars were in the same clade, while various brown cultivars were in a divergent clade [81, 82].

The highly restricted intron distribution patterns among cultivars maybe resulted from one of two possibly processes. Firstly, as mentioned above, despite the small number of samples, the distribution of IDP5 and IDP10 is similar to that of cultivated IDP3 and IDP11 (distributed in geographically areas), so we speculate that many strains containing different classes of IDP were cultivated early on, and that there might have been introgression of entire mitogenomes or sets of mitochondrial introns among these cultivars in mushroom farms. As a result, introns have been somewhat homogenized and all these cultivars have the same IDP. Second possibility is these cultivars were originally of the same mitochondrial genetic background and their original domesticated strains contained only these two IDPs. However, both hypothesis would suggest that these two IDPs were likely associated with a superior set of traits for mushroom cultivation. Indeed it has been experimentally shown that *A. bisporus* heterokaryons with different mitochondrial haplotypes had significantly different mycelial growth rates in the same nuclear background [83].

By comparing IDP3 and IDP11 with the rest of the IDPs within Clade Europe II and III, respectively, we found that both IDPs appear to have been formed by the loss of some introns that are present in many other IDPs. For example, IDP5 has one fewer intron (intron 25) than IDP6, while IDP3 has one fewer intron (intron 19) than IDP5. Similarly, in Clade Europe III, IDP11 has one fewer intron (intron 25) than IDP10. Our results also suggest that a specific intron missing from *cox1* has occurred widely in the domesticated strains, and this phenomenon is consistent with previous reports [84, 85]. Two hypotheses are proposed about how the *cox1* introns changed in domestication. One hypothesis is that the intron changes occurred prior to the domestication of *A. bisporus*. Many strains with different IDP classes were cultivated, and after artificial selection, some inferior IDP classes (5 and 10) were eliminated and superior IDP classes (3 and 11) were retained. In this scenario the two IDP classes (3 and 11) of cultivar-like wild strains served as ancestors for the selection and development of cultivars. The second hypothesis is that the *cox1* intron changed after the domestication of *A. bisporus*, primarily through intron loss. In Clade Europe II, the strains of IDP6 from Europe lost intron 25 to form IDP5, which is distributed in North America, and then continued to lose intron 19 to form the cultivar classes IDP3. This evolutionary pathway is consistent with historical records, which show that *A. bisporus* was originally grown in France and then spread to the United States, and that the strain contained in IDP3, “white” lineage, was discovered in the United States by the American mushroom farmer Lewis Downing in 1926 [86]. In Clade Europe III, the strain with IDP10 distributed in Europe lost intron 25 to form the cultivar classes IDP11.

The possible explanation for this phenomenon may be attributed to functional roles of introns as elucidated by investigations on eukaryotic nuclear gene. Currently known intron functions include three types: alternative splicing which can enhance proteome diversity [87], enhancing gene expression [88–90], and acting as various cis and trans regulatory elements [91, 92]. It’s possible that the introns within the mitochondrial genomes also have the above three types of functions. In addition, the complex environmental fluctuations that wild mushrooms face in natural environments could have favored a diversity of intron types and distributions. In contrast, the artificial culture environment for mushroom growing is relatively stable, nutrient rich, and homogeneous, which could have selected for the loss of introns in some of the genes as observed here.

The domestication and artificial selection for cultivars is a double-edged sword. On the one hand, high-performance strains are selected that meet human requirements in the standard mushroom growth model environment. On the other hand, such selected strains may be difficult to adapt to the multiple variable growth conditions in the natural environment, and the continued escape and invasion of domesticated germplasm into wild populations of this species can eventually lead to the loss of genetic diversity of *A. bisporus* populations. Our research provided an approaching that could detect the wild strains from cultivar-liked strains used the IDP. In this way, it is possible to use abundant wild germplasm resources to obtain new cultivar with excellent properties. In addition, some intron missing may lead to trait dominance, which can provide new ideas for future molecular breeding.

## Conclusions

In this study, we collected representative wild and cultivars strains of *A. bisporus* from around the world. The mitogenome of this species was investigated using a genome resequencing approach. Based on SNP data, all strains could be classified into seven genetically distinct and geographically associated clades, Clade Eurotetrasporus, Clade Europe I, Clade Europe II, Clade Europe III, Clade China, Clade America I, and Clade America II. Divergence time analysis indicated that the species formed in Europe 4.6 Ma and then spread eastward through eastern Asia (China) to North America. Since cultivars strains survive only in branches Clade Europe II and III, and detailed mitogenome structure studies indicate that differences in mitogenomes between clades are mainly due to insertions of *dpo* genes, we hypothesize that this may be a prerequisite for the domestication of strains from these two clades. Further studies showed that the *A. bisporus* population contains a total of 30 IDPs, while all cultivar strains contain only two IDPs (3 and 11), which clearly exhibit intron loss compared to the others, so we made two hypotheses (that the loss occurred before or after domestication). However, either hypothesis suggests that the change facilitates their adaptation to the cultivated environment.

This work introduces a model describing on mitogenome used *A. bisporus,* which insight into the evolutionary history of mushrooms from a new sight, and also provided a novel approaching on evaluation of germplasm resources in mushroom breeding.

## Supplementary Information

**Additional file 1:** **Supplementary Table S1.** Strain Information. **Supplementary Table S2.** IDP for each strain. **Supplementary Figure 1.** Mitogenome annotation for each strain. **Supplementary Figure 2.** Positions and distributions of SNPs in the reference genome. **Supplementary Figure 3.** Phylogenomic and population structure analysis. **Supplementary Figure 4.** CV error of each K values in population structure analysis. **Supplementary Figure 5.** Three evolutionary scenarios compared. **Supplementary Figure 6.** Projection of three scenarios on the first two LDA axes. **Supplementary Figure 7.** Different intron types at the same site within the cox1 gene.

## Data Availability

The raw sequence data reported in this paper have been deposited in the Genome Sequence Archive [93] in National Genomics Data Center [94], China National Center for Bioinformation / Beijing Institute of Genomics, Chinese Academy of Sciences (GSA: CRA009455) that are publicly accessible at https://ngdc.cncb.ac.cn/gsa. The mitogenome assembly data reported in this paper have been deposited in the GenBase in National Genomics Data Center [94], Beijing Institute of Genomics, Chinese Academy of Sciences/China National Center for Bioinformation, under accession number C_AA001640—C_AA002000 that is publicly accessible at https://ngdc.cncb.ac.cn/genbase.

## Abbreviations

MIR: A sequence in the middle of two inverted repeats sequences
IDP: Intron distribution pattern
IRS: A pair of inverted repeats sequences
Ma: Million years ago
