# Supplementary material for: Insight into the evolutionary and domesticated history of the most widely cultivated mushroom Agaricus bisporus via mitogenome sequences of 361 global strains
Source: BMC Genomics. 2023 Apr 5;24:182. doi: 10.1186/s12864-023-09257-w (PMC10077685; doi:10.1186/s12864-023-09257-w)
Supplement: Supplementary file 1 — Additional file 1: Supplementary Table S1. Strain Information. Supplementary Table S2. IDP for each strain. Supplementary Figure 1. Mitogenome annotation for each strain. Supplementary Figure 2. Positions and distributions of SNPs in the reference genome. Supplementary Figure 3. Phylogenomic and population structure analysis. Supplementary Figure 4. CV error of each K values in population structure analysis. Supplementary Figure 5. Three evolutionary scenarios compared. Supplementary Figure 6. Projection of three scenarios on the first two LDA axes. Supplementary Figure 7. Different intron types at the same site within the cox1 gene. [file 12864_2023_9257_MOESM1_ESM.pdf]

Supplementary Information

Multiple Clustal alignment of homing endonuclease genes of intron 23 of types 1, 2 and 3

```
intron23type1_BS603      IKIFEIYTFFNKNFDIEVSLLFSNFQPFLVNKIENSDNYLISEKNYIEPFFVGLLEGDGT
intron23type2_BS423      --TYLIDSMI--NFTIINKIIKRN--IYLKPSTEK-DRLNYVDQTYIEQFFVGLLEGDGT
intron23type3_BS033      --TYLI-LAS--NIIIADKILKSP-------------RIL-SD-TELGPFVVGLIDGDGS
                            : *      *: *  .::                .    : . :  *.***::***:
intron23type1_BS603      ITTDLN-SSSIRVRIIISLKNLPENVQMLKKIKETIGG--RVVIERKDKYATWIASNKKD
intron23type2_BS423      ITCSLIRKTTIFVRIIISLKNLPENLSMLTKIRDSIGG--RVIIEKKESYVTWIASNQKD
intron23type3_BS033      LQVNHWRKKNLQYRLVVKLADKPLNSDMLSHIAKVYGGYVRQGVEKESRYVQWI-INDQN
                         :  .   ...:  *:::.* : * * .**.:* .  **  *  :*::. *. **  *.::
intron23type1_BS603      LVK-ILAILA-KYPLLTARKQSQLEFAQDCLLNKDKDNYLINRKFKYKNKNLLLNNLAKL
intron23type2_BS423      LAK-VFVVLA-RYPLLTARKQCQLEFAKNCLLKRDLGNFNADRDNKYKNKILLLEDLSKR
intron23type3_BS033      TFKQTILPLLDNYPPLTSRMRLQYYFFKKFLQSPIVEHYFIERGLKYKNRDSILPLF--T
                           *  :  *  .** **:* : *  * :. * .    ::  :*  ****:  :*  :
intron23type1_BS603      ETLPFYFAPWLSGFIEAEGNFNLVFNEKGHLRKSSFSIGQNDELHILNWIKKYFNSNNSI
intron23type2_BS423      EL-PSYFAPWLSGFIEAEGNFSLVFNEKGQLRKSAFTIGQNDELHILKGIKSYFNSKNAI
intron23type3_BS033      EV-PNYFKEWLAGFIESEGSFSLRVQ--GN---YSFSIGQNHDCYLITAIRDYYGLSHLT
                         *  * **  **:****:**.*.* .:  *:    :*:****.: :::. *:.*:. .:
intron23type1_BS603      YTDKPKKNGNFQYYRLFLYNSDSRKHIFNHFLKYPLL-GYKKISFNNW--KIY-HESKSK
intron23type2_BS423      YKDKPKKGGNFEYYRLFLYNAESRKLLFEHFNKYPLL-GYKKLSYLNF--KNY---FDSK
intron23type3_BS033      IAYKTGKISGYPFYEFSVGSANGTGKVIDHC--TSLLQGYKYYQLAVFVSKNKVFNNRSK
                            *. * ..: :*.: : .::.   :::*    .** ***  .   :  *       **
intron23type1_BS603      Q
intron23type2_BS423      T
intron23type3_BS033      EFFE
```

>intron23type1_BS603
IKIFEIYTFFNKNFDIEVSLLFSNFQPFLVNKIENSDNYLISEKNYIEPFFVGLLEGDGTITTDLNSSSIRVRIIISLKNLPENVQ
MLKKIKETIGGRVVIERKDKYATWIASNKKDLVKILAILAKYPLLTARKQSQLEFAQDCLLNKDKDNYLINRKFKYKNK
NLLLNNLAKLETLPFYFAPWLSGFIEAEGNFNLVFNEKGHLRKSSFSIGQNDELHILNWIKKYFNSNNSIYTDKPKKNGN
FQYYRLFLYNSDSRKHIFNHFLKYPLLGYKKISFNNWKIYHESKSKQ
>intron23type2_BS423
TYLIDSMINFTIINKIIKRNIYLKPSTEKDRLNYVDQTYIEQFFVGLLEGDGTITCSLIRKTTIFVRIIISLKNLPENLSMLTKIR
DSIGGRVIIEKKESYVTWIASNQKDLAKVFVVLARYPLLTARKQCQLEFAKNCLLKRDLGNFNADRDNKYKNKILLLED
LSKRELPSYFAPWLSGFIEAEGNFSLVFNEKGQLRKSAFTIGQNDELHILKGIKSYFNSKNAIYKDKPKKGGNFEYYRLFL
YNAESRKLLFEHFNKYPLLGYKKLSYLNFKNYFDSKT
>intron23type3_BS033_cultivar_brown_in_our_study length 1121
TYLILASNIIIADKILKSPRILSDTELGPFVVGLIDGDGSLQVNHWRKKNLQYRLVVKLADKPLNSDMLSHIAKVYGGYV
RQGVEKESRYVQWIINDQNTFKQTILPLLDNYPPLTSRMRLQYYFFKKFLQSPIVEHYFIERGLKYKNRDSILPLFTEVPN
YFKEWLAGFIESEGSFSLRVQGNYSFSIGQNHDCYLITAIRDYYGLSHLTIAYKTGKISGYPFYEFSVGSANGTGKVIDHC
—TSLLQGYKYYQLAVFVSKNKVFNNRSKEFFE

Supplementary Table S1 Strain Information

Specimen	Taxon	Species and Clade	Original collection	Original number	ARP number	CIRM number	Country/ state-province-region-departement	Substrate of isolation	Habitat	BioProject	BioSample Accession	Experiment Accession	Assemble Accession
ZRL20160001	*A. sinodeliciosus*	A.sinodeliciosus	ZRL	-	-	-	China xinjiang	-	-	PRJCA014243	SAMC1038781	CRX570700	C AA001995.1
ARP276	A. cf. subfloccosus	A.subfloccosus	ARP	-	276	-	USA New mexico	-	-	PRJCA014243	SAMC1038782	CRX570701	C AA001965.1
QL20170217	*A. qilianensis*	A.qilianensis	ZRL	-	-	-	China qilian mountain	Litter	Picea crassifolia	PRJCA014243	SAMC1038783	CRX570702	C AA001983.1
ZRL20161973	*A. qilianensis*	A.qilianensis	ZRL	-	-	-	China qilian mountain	Litter	Picea crassifolia	PRJCA014243	SAMC1038784	CRX570703	C AA001999.1
QL20162067	*A. qilianensis*	A.qilianensis	ZRL	-	-	-	China qilian mountain	Litter	Picea crassifolia	PRJCA014243	SAMC1038785	CRX570704	C AA001980.1
ZRL20161862	*A. sinotetrasporus*	A.sinotetrasporus	ZRL	-	-	-	China qilian mountain	Litter	Picea crassifolia	PRJCA014243	SAMC1038786	CRX570705	C AA001998.1
QL20170258	*A. sinotetrasporus*	A.sinotetrasporus	ZRL	-	-	-	China qilian mountain	Litter	Picea crassifolia	PRJCA014243	SAMC1038787	CRX570706	C AA001984.1
QL20180590	*A. sinotetrasporus*	A.sinotetrasporus	ZRL	-	-	-	China qilian mountain	Litter	Picea crassifolia	PRJCA014243	SAMC1038788	CRX570707	C AA001986.1
ZRL20161020	*A. sinotetrasporus*	A.sinotetrasporus	ZRL	-	-	-	China qilian mountain	Litter	Picea crassifolia	PRJCA014243	SAMC1038789	CRX570708	C AA001996.1
BS423	*A. bisporus* var. *eurotetrasporus*	clade Eurotetrasporus	CGAB	-	-	2682	France/La Rochelle 85	Litter	C. macrocarpa	PRJCA014243	SAMC1038790	CRX570709	C AA001834.1
BS514	*A. bisporus* var. *eurotetrasporus*	clade Eurotetrasporus	CGAB	-	-	2681	Greece/Thessaly	Litter	Cupressus sempervirens	PRJCA014243	SAMC1038791	CRX570710	C AA001873.1
WTZ709-2048	A. bisporus var. bisporus	clade China	ZRL	-	-	-	China Tibet	-	-	PRJCA014243	SAMC1038792	CRX570711	C AA001994.1
ZRL20170159	A. bisporus var. bisporus	clade China	ZRL	-	-	-	China qilain mountian	Litter	Picea crassifolia	PRJCA014243	SAMC1038793	CRX570712	C AA002000.1
QL20170136	A. bisporus var. bisporus	clade China	ZRL	-	-	-	China qilain mountian	Litter	Picea crassifolia	PRJCA014243	SAMC1038794	CRX570713	C AA001981.1
ZRL20161813	A. bisporus var. bisporus	clade China	ZRL	-	-	-	China qilain mountian	Litter	Picea crassifolia	PRJCA014243	SAMC1038795	CRX570714	C AA001997.1
QL20170265	A. bisporus var. bisporus	clade China	ZRL	-	-	-	China qilain mountian	Litter	Picea crassifolia	PRJCA014243	SAMC1038796	CRX570715	C AA001985.1
QL20170216	A. bisporus var. bisporus	clade China	ZRL	-	-	-	China qilain mountian	Litter	Picea crassifolia	PRJCA014243	SAMC1038797	CRX570716	C AA001982.1
BS318-02	A. bisporus var. burnettii	clade America I	CGAB (ARP)	JB40-2	146	-	USA Californian Desert	Litter	Shrubs	PRJCA014243	SAMC1038798	CRX570717	C AA001795.1
BS311-04	A. bisporus var. burnettii	clade America I	CGAB (ARP)	JB33-4	139	-	USA Californian Desert	Litter	Shrubs	PRJCA014243	SAMC1038799	CRX570718	C AA001788.1
BS305-05	A. bisporus var. burnettii	clade America I	CGAB (ARP)	JB27-5	133	-	USA Californian Desert	Litter	Shrubs	PRJCA014243	SAMC1038800	CRX570719	C AA001783.1
BS728	A. bisporus var. burnettii	clade America I	RWK	JB146	-	-	USA Californian Desert	-	-	PRJCA014243	SAMC1038801	CRX570720	C AA001974.1
BS323-19	A. bisporus var. burnettii	clade America I	CGAB (ARP)	JB164-19	151	3192	USA Californian Desert	Litter	Shrubs	PRJCA014243	SAMC1038802	CRX570721	C AA001799.1
BS302-12	A. bisporus var. burnettii	clade America I	CGAB (ARP)	JB24-12	130	-	USA Californian Desert	Litter	Shrubs	PRJCA014243	SAMC1038803	CRX570722	C AA001779.1
BS309-03	A. bisporus var. burnettii	clade America I	CGAB (ARP)	JB31-3	137	-	USA Californian Desert	Litter	Shrubs	PRJCA014243	SAMC1038804	CRX570723	C AA001786.1
BS289-05	A. bisporus var. burnettii	clade America I	CGAB (ARP)	JB11-5	117	2697	USA Californian Desert	Litter	Shrubs	PRJCA014243	SAMC1038805	CRX570724	C AA001772.1
BS309-07	A. bisporus var. burnettii	clade America I	CGAB (ARP)	JB31-7	137	-	USA Californian Desert	Litter	Shrubs	PRJCA014243	SAMC1038806	CRX570725	C AA001787.1
BS302-11	A. bisporus var. burnettii	clade America I	CGAB (ARP)	JB24-11	130	-	USA Californian Desert	Litter	Shrubs	PRJCA014243	SAMC1038807	CRX570726	C AA001778.1
BS731	A. bisporus var. burnettii	clade America I	RWK	JB122		-	USA Californian Desert	-	-	PRJCA014243	SAMC1038808	CRX570727	C AA001975.1
BS327-8	A. bisporus var. burnettii	clade America I	CGAB (ARP)	JB157-8	155	-	USA Californian Desert	Litter	Shrubs	PRJCA014243	SAMC1038809	CRX570728	C AA001802.1
BS339-14	A. bisporus var. burnettii	clade America I	CGAB (ARP)	JB179-14	167	-	USA Californian Desert	Litter	Shrubs	PRJCA014243	SAMC1038810	CRX570729	C AA001809.1
BS317-01	A. bisporus var. burnettii	clade America I	CGAB (ARP)	JB39-1	145	-	USA Californian Desert	Litter	Shrubs	PRJCA014243	SAMC1038811	CRX570730	C AA001794.1
BS294-25	A. bisporus var. burnettii	clade America I	CGAB (ARP)	JB16-25	122	-	USA Californian Desert	Litter	Shrubs	PRJCA014243	SAMC1038812	CRX570731	C AA001774.1
BS290-5	A. bisporus var. burnettii	clade America I	CGAB (ARP)	JB12-5	118	-	USA Californian Desert	Litter	Shrubs	PRJCA014243	SAMC1038813	CRX570732	C AA001773.1
BS311-07	A. bisporus var. burnettii	clade America I	CGAB (ARP)	JB33-7	139	-	USA Californian Desert	Litter	Shrubs	PRJCA014243	SAMC1038814	CRX570733	C AA001789.1
BS303-08	A. bisporus var. burnettii	clade America I	CGAB (ARP)	JB25-8	131	-	USA Californian Desert	Litter	Shrubs	PRJCA014243	SAMC1038815	CRX570734	C AA001780.1
BS336-07	A. bisporus var. burnettii	clade America I	CGAB (ARP)	JB176-7	164	-	USA Californian Desert	Litter	Shrubs	PRJCA014243	SAMC1038816	CRX570735	C AA001808.1
BS306-01	A. bisporus var. burnettii	clade America I	CGAB (ARP)	JB28-1	134	-	USA Californian Desert	Litter	Shrubs	PRJCA014243	SAMC1038817	CRX570736	C AA001784.1
BS738	A. bisporus var. burnettii	clade America I	RWK	JB137		-	USA Californian Desert	-	-	PRJCA014243	SAMC1038818	CRX570737	C AA001978.1
BS314-04	A. bisporus var. burnettii	clade America I	CGAB (ARP)	JB36-4	142	-	USA Californian Desert	Litter	Shrubs	PRJCA014243	SAMC1038819	CRX570738	C AA001791.1
BS330-10	A. bisporus var. burnettii	clade America II	CGAB (ARP)	JB169-10	158	-	USA Californian Desert	Litter	Shrubs	PRJCA014243	SAMC1038820	CRX570739	C AA001804.1
BS330-06	A. bisporus var. burnettii	clade America II	CGAB (ARP)	JB169-6	158	-	USA Californian Desert	Litter	Shrubs	PRJCA014243	SAMC1038821	CRX570740	C AA001803.1
BS717	A. bisporus var. bisporus	clade America II	ARP	-	277	-	USA New mexico	-	-	PRJCA014243	SAMC1038822	CRX570741	C AA001966.1
BS713	A. bisporus var. bisporus	clade America II	ARP	-	269	-	USA New mexico	-	-	PRJCA014243	SAMC1038823	CRX570742	C AA001963.1
BS723	A. bisporus var. bisporus	clade America II	RWK	K1525	-	-	USA coastal Calif	-	-	PRJCA014243	SAMC1038824	CRX570743	C AA001971.1
BS142	A. bisporus var. bisporus	clade America II	ARP	FS-32	76	-	USA coastal Calif	Litter	C. macrocarpa	PRJCA014243	SAMC1038825	CRX570744	C AA001684.1
BS158	A. bisporus var. bisporus	clade America II	ARP	FS-58	102	-	USA coastal Calif	Litter	C. macrocarpa gelé et mort	PRJCA014243	SAMC1038826	CRX570745	C AA001694.1
BS590	A. bisporus var. bisporus	clade America II	ARP	AA0380	243	-	USA Calofornia	-	-	PRJCA014243	SAMC1038827	CRX570746	C AA001892.1
BS643	A. bisporus var. bisporus	clade America II	ARP	-	249	-	USA Calofornia	-	-	PRJCA014243	SAMC1038828	CRX570747	C AA001927.1
BS137	A. bisporus var. bisporus	clade America II	ARP	SM-1	67	-	USA coastal Calif	Litter	C. macrocarpa	PRJCA014243	SAMC1038829	CRX570748	C AA001681.1
BS711	A. bisporus var. bisporus	clade America II	ARP	KC-1	263	-	USA-Washington	Litter	Spruce	PRJCA014243	SAMC1038830	CRX570749	C AA001962.1
BS558	A. bisporus var. bisporus	clade America II	ARP	TF-14-2	239	-	USA coastal Calif	-	-	PRJCA014243	SAMC1038831	CRX570750	C AA001878.1
BS667	A. bisporus var. bisporus	clade America II	ARP	Sf 31	251	-	USA Calofornia	-	-	PRJCA014243	SAMC1038832	CRX570751	C AA001942.1
BS152	A. bisporus var. bisporus	clade America II	ARP	FS-44	88	-	USA coastal Calif	Litter	C. macrocarpa	PRJCA014243	SAMC1038833	CRX570752	C AA001690.1
BS109	A. bisporus var. bisporus	clade America II	ARP	BP-6	28	-	USA coastal Calif	Litter	C. macrocarpa	PRJCA014243	SAMC1038834	CRX570753	C AA001671.1
BS128	A. bisporus var. bisporus	clade America II	RWK	RWK 1291-67p	-	-	USA Calofornia	-	-	PRJCA014243	SAMC1038835	CRX570754	C AA001677.1
BS162	A. bisporus var. bisporus	clade America II	ARP	FS-62	106	-	USA coastal Calif	Litter	C. macrocarpa	PRJCA014243	SAMC1038836	CRX570755	C AA001696.1
BS165	A. bisporus var. bisporus	clade America II	ARP	FS-65	109	-	USA coastal Calif	Litter	C. macrocarpa	PRJCA014243	SAMC1038837	CRX570756	C AA001699.1
BS155	A. bisporus var. bisporus	clade America II	ARP	FS-49	93	-	USA coastal Calif	Litter	C. macrocarpa	PRJCA014243	SAMC1038838	CRX570757	C AA001692.1
BS734	A. bisporus var. burnettii	clade America II	RWK	JB128	-	-	USA Californian Desert	-	-	PRJCA014243	SAMC1038839	CRX570758	C AA001976.1
BS315-02	A. bisporus var. burnettii	clade America II	CGAB (ARP)	JB37-2	143	-	USA Californian Desert	Litter	Shrubs	PRJCA014243	SAMC1038840	CRX570759	C AA001792.1
BS297-09	A. bisporus var. burnettii	clade America II	CGAB (ARP)	JB19-9	125	-	USA Californian Desert	Litter	Shrubs	PRJCA014243	SAMC1038841	CRX570760	C AA001776.1
BS326-04	A. bisporus var. burnettii	clade America II	CGAB (ARP)	JB155-4	154	-	USA Californian Desert	Litter	Shrubs	PRJCA014243	SAMC1038842	CRX570761	C AA001801.1
BS316-03	A. bisporus var. burnettii	clade America II	CGAB (ARP)	JB38-3	144	-	USA Californian Desert	Litter	Shrubs	PRJCA014243	SAMC1038843	CRX570762	C AA001793.1
BS735	A. bisporus var. burnettii	clade America II	RWK	JB115	-	-	USA Californian Desert	-	-	PRJCA014243	SAMC1038844	CRX570763	C AA001977.1
BS304-01	A. bisporus var. burnettii	clade America II	CGAB (ARP)	JB26-1	132	-	USA Californian Desert	Litter	Shrubs	PRJCA014243	SAMC1038845	CRX570764	C AA001781.1
BS307-1	A. bisporus var. burnettii	clade America II	CGAB (ARP)	JB29-1	135	-	USA Californian Desert	Litter	Shrubs	PRJCA014243	SAMC1038846	CRX570765	C AA001785.1
BS304-09	A. bisporus var. burnettii	clade America II	CGAB (ARP)	JB26-9	132	-	USA Californian Desert	Litter	Shrubs	PRJCA014243	SAMC1038847	CRX570766	C AA001782.1
BS301-01	A. bisporus var. burnettii	clade America II	CGAB (ARP)	JB23-1	129	-	USA Californian Desert	Litter	Shrubs	PRJCA014243	SAMC1038848	CRX570767	C AA001777.1
BS324-01	A. bisporus var. burnettii	clade America II	CGAB (ARP)	JB165-1	152	-	USA Californian Desert	Litter	Shrubs	PRJCA014243	SAMC1038849	CRX570768	C AA001800.1
BS726	A. bisporus var. burnettii	clade America II	RWK	K1875	-	-	USA Californian Desert	-	-	PRJCA014243	SAMC1038850	CRX570769	C AA001973.1
BS320-06	A. bisporus var. burnettii	clade America II	CGAB (ARP)	JB161-6	148	-	USA Californian Desert	Litter	Shrubs	PRJCA014243	SAMC1038851	CRX570770	C AA001796.1
BS313-17	A. bisporus var. burnettii	clade America II	CGAB (ARP)	JB35-17	141	-	USA Californian Desert	Litter	Shrubs	PRJCA014243	SAMC1038852	CRX570771	C AA001790.1
BS331-08	A. bisporus var. burnettii	clade America II	CGAB (ARP)	JB171-8	159	-	USA Californian Desert	Litter	Shrubs	PRJCA014243	SAMC1038853	CRX570772	C AA001806.1
BS295-03	A. bisporus var. burnettii	clade America II	CGAB (ARP)	JB17-3	123	-	USA Californian Desert	Litter	Shrubs	PRJCA014243	SAMC1038854	CRX570773	C AA001775.1
BS214	A. bisporus var. bisporus	clade European I	CGAB	-	-	2721	France/Lorient 56	Litter	C. macrocarpa	PRJCA014243	SAMC1038855	CRX570774	C AA001724.1
BS426A	A. bisporus var. bisporus	clade European I	CGAB	-	-	2690	France/La Rochelle 85	Litter	C. macrocarpa	PRJCA014243	SAMC1038856	CRX570775	C AA001837.1
BS268	A. bisporus var. bisporus	clade European I	CGAB	-	-	2868	France/St Malo 35	Litter	C. macrocarpa	PRJCA014243	SAMC1038857	CRX570776	C AA001757.1
BS646	A. bisporus var. bisporus	clade European I	CGAB	-	-	2991	Greece/Thessaly	Manure	Goat or sheep de brebis	PRJCA014243	SAMC1038858	CRX570777	C AA001929.1
BS183	A. bisporus var. bisporus	clade European I	CGAB	-	-	2715	France/Lorient 56	Litter	C. macrocarpa	PRJCA014243	SAMC1038859	CRX570778	C AA001713.1
BS167A	A. bisporus var. bisporus	clade European I	CGAB	-	-	2757	France/Bordeaux 33	Litter	Cedrus	PRJCA014243	SAMC1038860	CRX570779	C AA001701.1
BS252	A. bisporus var. bisporus	clade European I	CGAB	-	-	2704	France/St Malo 22	Litter	C. macrocarpa	PRJCA014243	SAMC1038861	CRX570780	C AA001744.1
BS606	A. bisporus var. bisporus	clade European I	CGAB	-	-	2967	Greece/Thessaly	Litter	Cupressus semp.	PRJCA014243	SAMC1038862	CRX570781	C AA001907.1
BS213	A. bisporus var. bisporus	clade European I	CGAB	-	-	2720	France/Lorient 56	Litter	C. macrocarpa	PRJCA014243	SAMC1038863	CRX570782	C AA001723.1
BS722	A. bisporus var. bisporus	clade European I	CGAB	-	-	2842	France/11	-	-	PRJCA014243	SAMC1038864	CRX570783	C AA001970.1
BS369	A. bisporus var. bisporus	clade European I	CGAB	-	-	3100	France/St Malo 35	Litter	C. macrocarpa	PRJCA014243	SAMC1038865	CRX570784	C AA001823.1
BS581	A. bisporus var. bisporus	clade European I	CGAB	-	-	2963	France/ 86	Litter	Robinia pseudo-acacia	PRJCA014243	SAMC1038866	CRX570785	C AA001886.1
BS283B	A. bisporus var. bisporus	clade European I	CGAB	-	-	2872	France/La Rochelle 85	Litter	C. macrocarpa, Quercus	PRJCA014243	SAMC1038867	CRX570786	C AA001766.1
BS379	A. bisporus var. bisporus	clade European I	ARP	AMA-6	176	-	UK	Litter	-	PRJCA014243	SAMC1038868	CRX570787	C AA001828.1
BS368	A. bisporus var. bisporus	clade European I	CGAB	-	-	2823	France/St Malo 35	Litter	C. macrocarpa	PRJCA014243	SAMC1038869	CRX570788	C AA001822.1
BS249	A. bisporus var. bisporus	clade European I	CGAB	-	-	3162	France/St Malo 22	Litter	C. macrocarpa	PRJCA014243	SAMC1038870	CRX570789	C AA001741.1
BS218	A. bisporus var. bisporus	clade European I	CGAB	-	-	2906	France/Lorient 56	Litter	C. macrocarpa	PRJCA014243	SAMC1038871	CRX570790	C AA001726.1
BS554	A. bisporus var. bisporus	clade European I	CGAB	-	-	2956	France/La Rochelle 17	Litter	C. macrocarpa	PRJCA014243	SAMC1038872	CRX570791	C AA001877.1
BS199	A. bisporus var. bisporus	clade European I	CGAB	-	-	2725	France/Lorient 56	Litter	C. macrocarpa	PRJCA014243	SAMC1038873	CRX570792	C AA001716.1
BS282B	A. bisporus var. bisporus	clade European I	CGAB	-	-	2871	France/La Rochelle 85	Litter	C. macrocarpa, Quercus	PRJCA014243	SAMC1038874	CRX570793	C AA001765.1
BS253	A. bisporus var. bisporus	clade European I	CGAB	-	-	2703	France/St Malo 35	Litter	C. macrocarpa	PRJCA014243	SAMC1038875	CRX570794	C AA001745.1
BS429A	A. bisporus var. bisporus	clade European I	CGAB	-	-	2692	France/La Rochelle 85	Litter	C. macrocarpa	PRJCA014243	SAMC1038876	CRX570795	C AA001840.1
BS201	A. bisporus var. bisporus	clade European I	CGAB	-	-	2726	France/Lorient 56	Litter	C. macrocarpa	PRJCA014243	SAMC1038877	CRX570796	C AA001718.1
BS232	A. bisporus var. bisporus	clade European I	CGAB	-	-	2908	France/Lorient 56	Litter	C. macrocarpa	PRJCA014243	SAMC1038878	CRX570797	C AA001731.1
BS207	A. bisporus var. bisporus	clade European I	CGAB	-	-	2731	France/Lorient 56	Litter	C. macrocarpa	PRJCA014243	SAMC1038879	CRX570798	C AA001721.1
BS226	A. bisporus var. bisporus	clade European I	CGAB	-	-	2870	France/Lorient 56	Litter	C. macrocarpa	PRJCA014243	SAMC1038880	CRX570799	C AA001729.1
BS693	A. bisporus var. bisporus	clade European I	CGAB	-	-	3342	France/81	Litter	Populus	PRJCA014243	SAMC1038881	CRX570800	C AA001954.1
BS284B	A. bisporus var. bisporus	clade European I	CGAB	-	-	3172	France/La Rochelle 85	Litter	C. macrocarpa, Quercus	PRJCA014243	SAMC1038882	CRX570801	C AA001767.1
BS552	A. bisporus var. bisporus	clade European I	CGAB	-	-	2902	France/La Rochelle 17	Litter	C. macrocarpa	PRJCA014243	SAMC1038883	CRX570802	C AA001875.1
BS174	A. bisporus var. bisporus	clade European I	CGAB	-	-	2710	France/Lorient 56	Litter	C. macrocarpa	PRJCA014243	SAMC1038884	CRX570803	C AA001706.1
BS247	A. bisporus var. bisporus	clade European I	CGAB	-	-	3163	France/St Malo 22	Litter	C. macrocarpa	PRJCA014243	SAMC1038885	CRX570804	C AA001739.1
BS188	A. bisporus var. bisporus	clade European I	CGAB	-	-	2718	France/Lorient 56	Litter	Chamaecyparis	PRJCA014243	SAMC1038886	CRX570805	C AA001715.1
BS222	A. bisporus var. bisporus	clade European I	CGAB	-	-	3167	France/Lorient 56	Litter	C. macrocarpa	PRJCA014243	SAMC1038887	CRX570806	C AA001727.1
BS177	A. bisporus var. bisporus	clade European I	CGAB	-	-	2712	France/Lorient 56	Litter	C. macrocarpa	PRJCA014243	SAMC1038888	CRX570807	C AA001709.1
BS428B	A. bisporus var. bisporus	clade European I	CGAB	-	-	2691	France/La Rochelle 85	Litter	C. macrocarpa	PRJCA014243	SAMC1038889	CRX570808	C AA001839.1
BS180	A. bisporus var. bisporus	clade European I	CGAB	-	-	2801	France/Lorient 56	Litter	C. macrocarpa	PRJCA014243	SAMC1038890	CRX570809	C AA001711.1
BS425A	A. bisporus var. bisporus	clade European I	CGAB	-	-	3206	France/La Rochelle 85	Litter	C. macrocarpa	PRJCA014243	SAMC1038891	CRX570810	C AA001835.1
BS417	A. bisporus var. bisporus	clade European I	CGAB	-	-	3219	France/South-west 33	Litter	C. macrocarpa	PRJCA014243	SAMC1038892	CRX570811	C AA001831.1
BS553	A. bisporus var. bisporus	clade European I	CGAB	-	-	2892	France/La Rochelle 17	Litter	C. macrocarpa	PRJCA014243	SAMC1038893	CRX570812	C AA001876.1
BS235	A. bisporus var. bisporus	clade European I	CGAB	-	-	2835	France/Lorient 56	Litter	C. macrocarpa	PRJCA014243	SAMC1038894	CRX570813	C AA001733.1
BS212	A. bisporus var. bisporus	clade European I	CGAB	-	-	2719	France/Lorient 56	Litter	C. macrocarpa	PRJCA014243	SAMC1038895	CRX570814	C AA001722.1
BS422B	A. bisporus var. bisporus	clade European I	CGAB	-	-	2897	France/South-west 33	Litter	C. macrocarpa	PRJCA014243	SAMC1038896	CRX570815	C AA001833.1
BS596	A. bisporus var. bisporus	clade European I	CGAB	-	-	3103	Greece/Island of Evia	Litter	Cupressus semp.	PRJCA014243	SAMC1038897	CRX570816	C AA001897.1
BS672	A. bisporus var. bisporus	clade European I	CGAB	-	-	2938	Greece/Thessaly	Litter	C. sempervirens	PRJCA014243	SAMC1038898	CRX570817	C AA001944.1
BS597	A. bisporus var. bisporus	clade European I	CGAB	-	-	2975	Greece/Island of Evia	Litter	Cupressus semp.	PRJCA014243	SAMC1038899	CRX570818	C AA001898.1
BS070D	A. bisporus var. bisporus	clade European I	CGAB	-	-	2952	France/ 44	Manure	Goat or sheep	PRJCA014243	SAMC1038900	CRX570819	C AA001657.1
BS652	A. bisporus var. bisporus	clade European I	CGAB	-	-	2925	Greece/Thessaly	Litter	Cupressus semp.	PRJCA014243	SAMC1038901	CRX570820	C AA001933.1
BS612	A. bisporus var. bisporus	clade European I	CGAB	-	-	2986	France/Bordeaux 33	Litter	Cupressocyparis	PRJCA014243	SAMC1038902	CRX570821	C AA001911.1
BS450	A. bisporus var. bisporus	clade European I	CGAB	-	-	3275	France/Bordeaux 33	Manure	Goat or sheep	PRJCA014243	SAMC1038903	CRX570822	C AA001853.1
BS078E	A. bisporus var. bisporus	clade European I	CGAB	-	-	2740	France/Bordeaux 33	Manure	Goat or sheep	PRJCA014243	SAMC1038904	CRX570823	C AA001664.1
BS341A	A. bisporus var. bisporus	clade European I	CGAB	-	-	2860	France/Lorient 56	Litter	C. macrocarpa	PRJCA014243	SAMC1038905	CRX570824	C AA001810.1
BS585	A. bisporus var. bisporus	clade European I	RWK	I1	-	-	Israël	-	-	PRJCA014243	SAMC1038906	CRX570825	C AA001888.1
BS587	A. bisporus var. bisporus	clade European I	ARP	I4	16	-	Israël	Litter	Eucalyptus	PRJCA014243	SAMC1038907	CRX570826	C AA001889.1
BS645	A. bisporus var. bisporus	clade European I	CGAB	-	-	2920	Greece/Thessaly	Manure	Goat or sheep	PRJCA014243	SAMC1038908	CRX570827	C AA001928.1
BS656	A. bisporus var. bisporus	clade European I	CGAB	-	-	2921	Greece/Thessaly	Litter	Cupressus semp.	PRJCA014243	SAMC1038909	CRX570828	C AA001934.1
BS631	A. bisporus var. bisporus	clade European I	CGAB	-	-	2943	Greece/Island of Evia	Litter	Cupressus semp.	PRJCA014243	SAMC1038910	CRX570829	C AA001923.1
BS651	A. bisporus var. bisporus	clade European I	CGAB	-	-	3344	Greece/Thessaly	Litter	Cupressus semp.	PRJCA014243	SAMC1038911	CRX570830	C AA001932.1
BS662	A. bisporus var. bisporus	clade European I	CGAB	-	-	2926	Greece/Peloponese	Litter	Cupressus semp.	PRJCA014243	SAMC1038912	CRX570831	C AA001937.1
BS666	A. bisporus var. bisporus	clade European I	CGAB	-	-	2930	Greece/Peloponese	Litter	Cupressus semp.	PRJCA014243	SAMC1038913	CRX570832	C AA001941.1
BS628	A. bisporus var. bisporus	clade European I	CGAB	-	-	2944	Greece/Island of Evia	Litter	Cupressus semp.	PRJCA014243	SAMC1038914	CRX570833	C AA001922.1
BS665	A. bisporus var. bisporus	clade European I	CGAB	-	-	3000	Greece/Peloponese	Litter	Cupressus semp.	PRJCA014243	SAMC1038915	CRX570834	C AA001940.1
BS663	A. bisporus var. bisporus	clade European I	CGAB	-	-	2972	Greece/Peloponese	Litter	Cupressus semp.	PRJCA014243	SAMC1038916	CRX570835	C AA001938.1
BS203	A. bisporus var. bisporus	clade European I	CGAB	-	-	2728	France/Lorient 56	Litter	C. macrocarpa	PRJCA014243	SAMC1038917	CRX570836	C AA001720.1
BS601	A. bisporus var. bisporus	clade European I	CGAB	-	-	3326	Greece/Thessaly	Litter	Cupressus semp.	PRJCA014243	SAMC1038918	CRX570837	C AA001902.1
BS698	A. bisporus var. bisporus	clade European I	Diakov/Lidia V. Marina	VKMF 1577	-	2826	Russia/Altay Republic	Litter	Meadow under Cedrus	PRJCA014243	SAMC1038919	CRX570838	C AA001958.1
BS725	A. bisporus var. bisporus	clade European I	RWK	K1634	-	-	Canada-Alberta	-	-	PRJCA014243	SAMC1038920	CRX570839	C AA001972.1
BS511	A. bisporus var. bisporus	clade European I	CGAB	-	-	2890	Greece/Thessaly	Manure	Goat or sheep	PRJCA014243	SAMC1038921	CRX570840	C AA001870.1
BS510	A. bisporus var. bisporus	clade European I	CGAB	-	-	2880	Greece/Thessaly	Manure	Goat or sheep	PRJCA014243	SAMC1038922	CRX570841	C AA001869.1
BS269	A. bisporus var. bisporus	clade European I	CGAB	-	-	2883	France/St Malo 35	Litter	C. macrocarpa	PRJCA014243	SAMC1038923	CRX570842	C AA001758.1
BS358	A. bisporus var. bisporus	clade European I	CGAB	-	-	2830	France/St Malo 35	Litter	C. macrocarpa	PRJCA014243	SAMC1038924	CRX570843	C AA001814.1
BS262	A. bisporus var. bisporus	clade European I	CGAB	-	-	2865	France/St Malo 35	Litter	C. macrocarpa	PRJCA014243	SAMC1038925	CRX570844	C AA001752.1
BS365	A. bisporus var. bisporus	clade European I	CGAB	-	-	2816	France/St Malo 35	Litter	C. macrocarpa	PRJCA014243	SAMC1038926	CRX570845	C AA001818.1
BS359	A. bisporus var. bisporus	clade European I	CGAB	-	-	2808	France/St Malo 35	Litter	C. macrocarpa	PRJCA014243	SAMC1038927	CRX570846	C AA001815.1
BS267	A. bisporus var. bisporus	clade European I	CGAB	-	-	2867	France/St Malo 35	Litter	C. macrocarpa	PRJCA014243	SAMC1038928	CRX570847	C AA001756.1
BS361	A. bisporus var. bisporus	clade European I	CGAB	-	-	2813	France/St Malo 35	Litter	C. macrocarpa	PRJCA014243	SAMC1038929	CRX570848	C AA001816.1
BS202A	A. bisporus var. bisporus	clade European I	CGAB	-	-	2727	France/Lorient 56	Litter	C. macrocarpa	PRJCA014243	SAMC1038930	CRX570849	C AA001719.1
BS425D	A. bisporus var. bisporus	clade European I	CGAB	-	-	3209	France/La Rochelle 85	Litter	C. macrocarpa	PRJCA014243	SAMC1038931	CRX570850	C AA001836.1
BS432B	A. bisporus var. bisporus	clade European I	CGAB	-	-	3244	France/La Rochelle 17	Litter	C. macrocarpa	PRJCA014243	SAMC1038932	CRX570851	C AA001843.1
BS286C	A. bisporus var. bisporus	clade European I	CGAB	-	-	2818	France/South-west 33	Litter	C. macrocarpa	PRJCA014243	SAMC1038933	CRX570852	C AA001769.1
BS217	A. bisporus var. bisporus	clade European I	CGAB	-	-	2905	France/Lorient 56	Litter	C. macrocarpa	PRJCA014243	SAMC1038935	CRX570854	C AA001725.1
BS432A	A. bisporus var. bisporus	clade European I	CGAB	-	-	2810	France/La Rochelle 17	Litter	C. macrocarpa	PRJCA014243	SAMC1038936	CRX570855	C AA001842.1
BS433A	A. bisporus var. bisporus	clade European I	CGAB	-	-	2809	France/La Rochelle 17	Litter	C. macrocarpa	PRJCA014243	SAMC1038937	CRX570856	C AA001844.1
BS426D	A. bisporus var. bisporus	clade European I	CGAB	-	-	2895	France/La Rochelle 85	Litter	C. macrocarpa	PRJCA014243	SAMC1038938	CRX570857	C AA001838.1
BS497	A. bisporus var. bisporus	clade European I	CGAB	-	-	2879	Greece/Thessaly	Manure	Goat or sheep	PRJCA014243	SAMC1038939	CRX570858	C AA001865.1
BS577	A. bisporus var. bisporus	clade European I	CGAB	-	-	2987	France/South-west 33	Litter	C. macrocarpa	PRJCA014243	SAMC1038940	CRX570859	C AA001884.1
BS363	A. bisporus var. bisporus	clade European II	CGAB	-	-	2807	France/St Malo 35	Litter	C. macrocarpa	PRJCA014243	SAMC1038941	CRX570860	C AA001817.1
BS059	A. bisporus var. bisporus	clade European II	Le Lion B92	-	-	3201	Cultivar white	-	-	PRJCA014243	SAMC1038942	CRX570861	C AA001654.1
BS460	A. bisporus var. bisporus	clade European II	RWK/Loftus	FS17A	-	-	USA coastal Calif	-	-	PRJCA014243	SAMC1038943	CRX570862	C AA001855.1
3X	A. bisporus var. bisporus	clade European II	ZRL	-	-	-	Cultivated in China	-	-	PRJCA014243	SAMC1038944	CRX570863	C AA001640.1
BS026-7	A. bisporus var. bisporus	clade European II	-	U1-7	-	2982	Cultivar hybrid	-	-	PRJCA014243	SAMC1038945	CRX570864	C AA001649.1
BS574	A. bisporus var. bisporus	clade European II	CGAB	-	-	2694	Greece/Thessaly	Manure	Goat or sheep	PRJCA014243	SAMC1038946	CRX570865	C AA001883.1
BS492	A. bisporus var. bisporus	clade European II	CGAB	-	-	3281	Greece/Thessaly	Litter	Cupressus sempervirens	PRJCA014243	SAMC1038947	CRX570866	C AA001862.1
BS573	A. bisporus var. bisporus	clade European II	CGAB	-	-	2965	Greece/Thessaly	Manure	Goat or sheep	PRJCA014243	SAMC1038948	CRX570867	C AA001882.1
BS506	A. bisporus var. bisporus	clade European II	CGAB	-	-	2862	Greece/Thessaly	Manure	Goat or sheep	PRJCA014243	SAMC1038949	CRX570868	C AA001867.1
BS118H	A. bisporus var. bisporus	clade European II	CGAB	-	-	2769	France/71	Litter	Ensilage, foin	PRJCA014243	SAMC1038950	CRX570869	C AA001676.1
BS071A	A. bisporus var. bisporus	clade European II	CGAB	-	-	3186	France/ 44	Manure	Goat or sheep	PRJCA014243	SAMC1038951	CRX570870	C AA001658.1
WTZ694-2090	A. bisporus var. bisporus	clade European II	ZRL	-	-	-	China Tibet	-	-	PRJCA014243	SAMC1038952	CRX570871	C AA001993.1
BS321-11	A. bisporus var. burnettii	clade European II	CGAB (ARP)	JB162-11	149	-	USA Californian Desert	Litter	Shrubs	PRJCA014243	SAMC1038953	CRX570872	C AA001797.1
BS321	A. bisporus var. burnettii	clade European II	CGAB (ARP)	JB162-11	149	-	USA Californian Desert	Litter	Shrubs	PRJCA014243	SAMC1038954	CRX570873	C AA001798.1
BS475	A. bisporus var. bisporus	clade European II	Diakov/ARP	47P	222	3258	Russia/Penza	-	-	PRJCA014243	SAMC1038955	CRX570874	C AA001858.1
BS479	A. bisporus var. bisporus	clade European II	Diakov/ARP	53P	226	-	Russia/Penza	-	-	PRJCA014243	SAMC1038956	CRX570875	C AA001860.1
BS715	A. bisporus var. bisporus	clade European II	ARP	-	274	-	USA New mexico	-	-	PRJCA014243	SAMC1038957	CRX570876	C AA001964.1
BS465	A. bisporus var. bisporus	clade European II	ARP	ROH-1	212	-	USA New mexico	-	-	PRJCA014243	SAMC1038958	CRX570877	C AA001857.1
BS703	A. bisporus var. bisporus	clade European II	CGAB	-	-	2837	France/La Rochelle 17	-	-	PRJCA014243	SAMC1038959	CRX570878	C AA001960.1
BS617	A. bisporus var. bisporus	clade European II	CGAB	-	-	3329	Greece/Thessaly	Litter	Cupressus semp.	PRJCA014243	SAMC1038960	CRX570879	C AA001915.1
BS509	A. bisporus var. bisporus	clade European II	CGAB	-	-	2915	Greece/Thessaly	Manure	Goat or sheep	PRJCA014243	SAMC1038961	CRX570880	C AA001868.1
BS134	A. bisporus var. bisporus	clade European II	ARP	JP-1	56	-	Canada-Ontario	Litter	Trail	PRJCA014243	SAMC1038962	CRX570881	C AA001678.1
BS676	A. bisporus var. bisporus	clade European II	CGAB	-	-	3262	Greece/Thessaly	Sheep manure	Goat or sheep	PRJCA014243	SAMC1038963	CRX570882	C AA001947.1
BS709	A. bisporus var. bisporus	clade European II	ARP	KP-2	261	-	USA-Massachussetts	-	-	PRJCA014243	SAMC1038964	CRX570883	C AA001961.1
BS377	A. bisporus var. bisporus	clade European II	ARP	AMA-4	174	-	UK	Litter	Ulmus, Crataegus	PRJCA014243	SAMC1038965	CRX570884	C AA001826.1
BS034	A. bisporus var. bisporus	clade European II	Somycel/Sylvan 53	-	-	3202	Cultivar white	-	-	PRJCA014243	SAMC1038966	CRX570885	C AA001652.1
BS154	A. bisporus var. bisporus	clade European II	ARP	FS-48	92	-	USA coastal Calif	Litter	C. macrocarpa	PRJCA014243	SAMC1038967	CRX570886	C AA001691.1
BS694	A. bisporus var. bisporus	clade European II	G Mata	RG-IE176		2840	Mexico Veracruz	Litter	C. benthamii var. lindleyi	PRJCA014243	SAMC1038968	CRX570887	C AA001955.1
BS110	A. bisporus var. bisporus	clade European II	ARP	FS-12	29	-	USA coastal Calif	Litter	C. macrocarpa, Pinus	PRJCA014243	SAMC1038969	CRX570888	C AA001672.1
BS687	A. bisporus var. bisporus	clade European II	PC-G Mata	IE623		3188	Mexico Tlaxcala	Litter	C. benthamii var. lindleyi	PRJCA014243	SAMC1038970	CRX570889	C AA001951.1
BS071B	A. bisporus var. bisporus	clade European II	CGAB	-		2961	France/ 44	Manure	Goat or sheep	PRJCA014243	SAMC1038971	CRX570890	C AA001659.1
BS334-12	A. bisporus var. burnettii	clade European II	CGAB (ARP)	JB174-12	162	-	USA Californian Desert	Litter	Shrubs	PRJCA014243	SAMC1038972	CRX570891	C AA001807.1
BS135	A. bisporus var. bisporus	clade European II	ARP	BP-7	64	-	USA coastal Calif	Litter	Pinus, Picea	PRJCA014243	SAMC1038973	CRX570892	C AA001679.1
As2796	A. bisporus var. bisporus	clade European II	ZRL	-	-	-	Cultivated in China	-	-	PRJCA014243	SAMC1038974	CRX570893	C AA001643.1
BS140	A. bisporus var. bisporus	clade European II	ARP	FS-27	71	-	USA coastal Calif	Litter	C. macrocarpa	PRJCA014243	SAMC1038975	CRX570894	C AA001683.1
BS588	A. bisporus var. bisporus	clade European II	ARP	W699	241	-	UK	Litter	Linum	PRJCA014243	SAMC1038976	CRX570895	C AA001890.1
BS275	A. bisporus var. bisporus	clade European II	CGAB	-	-	2884	France/St Malo 35	Litter	Chamaecyparis	PRJCA014243	SAMC1038977	CRX570896	C AA001761.1
BS355	A. bisporus var. bisporus	clade European II	CGAB	-	-	2803	France/St Malo 35	Litter	C. macrocarpa	PRJCA014243	SAMC1038978	CRX570897	C AA001812.1
BS274	A. bisporus var. bisporus	clade European II	CGAB	-	-	2882	France/St Malo 35	Litter	C. macrocarpa	PRJCA014243	SAMC1038979	CRX570898	C AA001760.1
BS258	A. bisporus var. bisporus	clade European II	CGAB	-	-	2686	France/St Malo 35	Litter	C. macrocarpa	PRJCA014243	SAMC1038980	CRX570899	C AA001749.1
BS164	A. bisporus var. bisporus	clade European II	ARP	FS-64	108	-	USA coastal Calif	Litter, Manure	C. macrocarpa; Goat or sheep	PRJCA014243	SAMC1038981	CRX570900	C AA001698.1
BS276	A. bisporus var. bisporus	clade European II	CGAB	-	-	2685	France/St Malo 35	Litter	C. macrocarpa	PRJCA014243	SAMC1038982	CRX570901	C AA001762.1
BS639	A. bisporus var. bisporus	clade European II	ARP	-	245	-	UK	-	-	PRJCA014243	SAMC1038983	CRX570902	C AA001925.1
BS104	A. bisporus var. bisporus	clade European II	ARP	FS-20	40	-	USA coastal Calif	Litter	C. macrocarpa	PRJCA014243	SAMC1038984	CRX570903	C AA001669.1
BS103	A. bisporus var. bisporus	clade European II	ARP	FS-19	39	-	USA coastal Calif	Litter	C. macrocarpa	PRJCA014243	SAMC1038985	CRX570904	C AA001668.1
BS696	A. bisporus var. bisporus	clade European II	PC-G Mata	M 674		2864	Mexico Veracruz	Litter	C. benthamii var. lindleyi	PRJCA014243	SAMC1038986	CRX570905	C AA001956.1
W192	A. bisporus var. bisporus	clade European II	ZRL	-	-	-	Cultivated in China	-	-	PRJCA014243	SAMC1038987	CRX570906	C AA001990.1
BS139	A. bisporus var. bisporus	clade European II	ARP	FS-26	70	-	USA coastal Calif	Litter	Sparse grass	PRJCA014243	SAMC1038988	CRX570907	C AA001682.1
901	A. bisporus var. bisporus	clade European II	ZRL	-	-	-	Cultivated in China	-	-	PRJCA014243	SAMC1038989	CRX570908	C AA001641.1
W38	A. bisporus var. bisporus	clade European II	ZRL	-	-	-	Cultivated in China	-	-	PRJCA014243	SAMC1038990	CRX570909	C AA001992.1
BS114	A. bisporus var. bisporus	clade European II	ARP	FS-15	35	-	USA coastal Calif	Litter	C. macrocarpa	PRJCA014243	SAMC1038991	CRX570910	C AA001675.1
BS021	A. bisporus var. bisporus	clade European II	Somycel/Sylvan 91/191	-	-	2699	Cultivar white	-	-	PRJCA014243	SAMC1038992	CRX570911	C AA001647.1
BS375	A. bisporus var. bisporus	clade European II	ARP	GW-1	171	-	USA-Washington	-	-	PRJCA014243	SAMC1038993	CRX570912	C AA001825.1
U1	A. bisporus var. bisporus	clade European II	ZRL	-	-	2982	Cultivated in China	-	-	PRJCA014243	SAMC1038994	CRX570913	C AA001987.1
BS403	A. bisporus var. bisporus	clade European II	Somycel/Sylvan 608	-	-	3223	Cultivar hybrid	-	-	PRJCA014243	SAMC1038995	CRX570914	C AA001829.1
A15	A. bisporus var. bisporus	clade European II	ZRL	-	-	-	Cultivated in China	-	-	PRJCA014243	SAMC1038996	CRX570915	C AA001642.1
W2000	A. bisporus var. bisporus	clade European II	ZRL	-	-	-	Cultivated in China	-	-	PRJCA014243	SAMC1038997	CRX570916	C AA001991.1
BS224	A. bisporus var. bisporus	clade European III	CGAB	-	-	2869	France/Lorient 56	Litter	C. macrocarpa	PRJCA014243	SAMC1038998	CRX570917	C AA001728.1

Supplementary Table S1 Continued.

Specimen	Taxon	Species and Clade	Original collection	Original number	ARP number	CIRM number	Country/ state-province-region-departement	Substrate of isolation	Habitat	BioProject	BioSample Accession	Experiment Accession	Assemble Accession
BS595	A. bisporus var. bisporus	clade European III	CGAB	-	-	3330	Greece/Island of Evia	Litter	Cupressus semp.	PRJCA014243	SAMC1038999	CRX570918	C AA001896.1
BS594	A. bisporus var. bisporus	clade European III	CGAB	-	-	3331	Greece/Island of Evia	Litter	Cupressus semp.	PRJCA014243	SAMC1039000	CRX570919	C AA001895.1
BS285	A. bisporus var. bisporus	clade European III	CGAB	-	-	2828	France/La Rochelle 85	Litter	C. macrocarpa, Quercus	PRJCA014243	SAMC1039001	CRX570920	C AA001768.1
BS243	A. bisporus var. bisporus	clade European III	CGAB	-	-	3171	France/Lorient 56	Litter	C. macrocarpa	PRJCA014243	SAMC1039002	CRX570921	C AA001737.1
BS650	A. bisporus var. bisporus	clade European III	CGAB	-	-	2923	Greece/Thessaly	Litter	Cupressus semp.	PRJCA014243	SAMC1039003	CRX570922	C AA001931.1
BS593	A. bisporus var. bisporus	clade European III	CGAB	-	-	2977	Greece/Island of Evia	Litter	Cupressus semp.	PRJCA014243	SAMC1039004	CRX570923	C AA001894.1
BS603	A. bisporus var. bisporus	clade European III	CGAB	-	-	3289	Greece/Thessaly	Litter	Cupressus semp.	PRJCA014243	SAMC1039005	CRX570924	C AA001904.1
BS622	A. bisporus var. bisporus	clade European III	CGAB	-	-	2939	Greece/Thessaly	Litter	Cupressus semp.	PRJCA014243	SAMC1039006	CRX570925	C AA001919.1
BS602	A. bisporus var. bisporus	clade European III	CGAB	-	-	3327	Greece/Thessaly	Litter	Cupressus semp.	PRJCA014243	SAMC1039007	CRX570926	C AA001903.1
BS416C	A. bisporus var. bisporus	clade European III	CGAB	-	-	2914	France/ 86	Litter	Robinia, Sparse grass	PRJCA014243	SAMC1039008	CRX570927	C AA001830.1
BS560	A. bisporus var. bisporus	clade European III	CGAB	-	-	3333	Greece/Island of Evia	Litter	Cupressus semp.	PRJCA014243	SAMC1039009	CRX570928	C AA001879.1
BS089	A. bisporus var. bisporus	clade European III	PSUMCC	-	-	3194	Cultivar golden white	-	-	PRJCA014243	SAMC1039010	CRX570929	C AA001666.1
BS078A	A. bisporus var. bisporus	clade European III	CGAB	-	-	2736	France/Bordeaux 33	Manure	Goat or sheep	PRJCA014243	SAMC1039011	CRX570930	C AA001663.1
BS025	A. bisporus var. bisporus	clade European III	Somycel/Sylvan 76/676	-	-	3197	Cultivar off white	-	-	PRJCA014243	SAMC1039012	CRX570931	C AA001648.1
BS551	A. bisporus var. bisporus	clade European III	Somycel/Sylvan U3	-	-	2700	Cultivar hybrid	-	-	PRJCA014243	SAMC1039013	CRX570932	C AA001874.1
U3	A. bisporus var. bisporus	clade European III	ZRL	-	-	-	Cultivated in China	-	-	PRJCA014243	SAMC1039014	CRX570933	C AA001988.1
W176	A. bisporus var. bisporus	clade European III	ZRL	-	-	-	Cultivated in China	-	-	PRJCA014243	SAMC1039015	CRX570934	C AA001989.1
BS170	A. bisporus var. bisporus	clade European III	CGAB	-	-	2773	France/ 53	Manure	Goat or sheep	PRJCA014243	SAMC1039016	CRX570935	C AA001703.1
BS330-6	A. bisporus var. burnettii	clade European III	CGAB (ARP)	JB169-6	158	-	USA Californian Desert	Litter	Shrubs	PRJCA014243	SAMC1039017	CRX570936	C AA001805.1
BS449	A. bisporus var. bisporus	clade European III	CGAB	-	-	2934	France/Bordeaux 33	Manure	Goat or sheep	PRJCA014243	SAMC1039018	CRX570937	C AA001852.1
BS719	A. bisporus var. bisporus	clade European III	CGAB	-	-	2877	France/61	-	-	PRJCA014243	SAMC1039019	CRX570938	C AA001968.1
BS431A	A. bisporus var. bisporus	clade European III	CGAB	-	-	3101	France/La Rochelle 17	Litter	C. macrocarpa	PRJCA014243	SAMC1039020	CRX570939	C AA001841.1
BS136	A. bisporus var. bisporus	clade European III	ARP	BP-8	65	-	USA coastal Calif	Litter	Picea	PRJCA014243	SAMC1039021	CRX570940	C AA001680.1
BS679	A. bisporus var. bisporus	clade European III	CGAB	-	-	3346	Spain/Aragon	Litter	C. sempervirens	PRJCA014243	SAMC1039022	CRX570941	C AA001948.1
BS228	A. bisporus var. bisporus	clade European III	CGAB	-	-	3205	France/Lorient 56	Litter	C. macrocarpa	PRJCA014243	SAMC1039023	CRX570942	C AA001730.1
BS265	A. bisporus var. bisporus	clade European III	CGAB	-	-	2886	France/St Malo 35	Litter	C. macrocarpa	PRJCA014243	SAMC1039024	CRX570943	C AA001754.1
BS721	A. bisporus var. bisporus	clade European III	CGAB	-	-	3352	France/South-west 40	Litter	-	PRJCA014243	SAMC1039025	CRX570944	C AA001969.1
BS145	A. bisporus var. bisporus	clade European III	ARP	FS-36	80	-	USA coastal Calif	Litter	C. macrocarpa	PRJCA014243	SAMC1039026	CRX570945	C AA001686.1
BS366B	A. bisporus var. bisporus	clade European III	CGAB	-	-	3216	France/Bordeaux 33	Litter	-	PRJCA014243	SAMC1039027	CRX570946	C AA001819.1
BS633	A. bisporus var. bisporus	clade European III	Geml	NV	-	-	Hungria	-	-	PRJCA014243	SAMC1039028	CRX570947	C AA001924.1
BS075A	A. bisporus var. bisporus	clade European III	CGAB	-	-	2753	France/Bordeaux 33	Litter	Picea	PRJCA014243	SAMC1039029	CRX570948	C AA001660.1
BS178	A. bisporus var. bisporus	clade European III	CGAB	-	-	2713	France/Lorient 56	Litter	C. macrocarpa	PRJCA014243	SAMC1039030	CRX570949	C AA001710.1
BS264	A. bisporus var. bisporus	clade European III	CGAB	-	-	2866	France/St Malo 35	Litter	C. macrocarpa	PRJCA014243	SAMC1039031	CRX570950	C AA001753.1
BS378	A. bisporus var. bisporus	clade European III	ARP	AMA-5	175	-	UK	Litter	Conifers	PRJCA014243	SAMC1039032	CRX570951	C AA001827.1
BS259	A. bisporus var. bisporus	clade European III	CGAB	-	-	2911	France/St Malo 35	Litter	C. macrocarpa	PRJCA014243	SAMC1039033	CRX570952	C AA001750.1
BS437A	A. bisporus var. bisporus	clade European III	CGAB	-	-	2822	France/La Rochelle 17	Litter	C. macrocarpa	PRJCA014243	SAMC1039034	CRX570953	C AA001848.1
BS181	A. bisporus var. bisporus	clade European III	CGAB	-	-	2714	France/Lorient 56	Litter	C. macrocarpa	PRJCA014243	SAMC1039035	CRX570954	C AA001712.1
BS233	A. bisporus var. bisporus	clade European III	CGAB	-	-	2909	France/Lorient 56	Litter	C. macrocarpa	PRJCA014243	SAMC1039036	CRX570955	C AA001732.1
BS266	A. bisporus var. bisporus	clade European III	CGAB	-	-	2936	France/St Malo 35	Litter	C. macrocarpa	PRJCA014243	SAMC1039037	CRX570956	C AA001755.1
BS105	A. bisporus var. bisporus	clade European III	ARP	FS-21	41	-	USA coastal Calif	Litter	C. macrocarpa	PRJCA014243	SAMC1039038	CRX570957	C AA001670.1
BS150	A. bisporus var. bisporus	clade European III	ARP	FS-41	85	-	USA coastal Calif	Litter	C. macrocarpa	PRJCA014243	SAMC1039039	CRX570958	C AA001689.1
BS357	A. bisporus var. bisporus	clade European III	CGAB	-	-	2831	France/St Malo 35	Litter	C. macrocarpa	PRJCA014243	SAMC1039040	CRX570959	C AA001813.1
BS176	A. bisporus var. bisporus	clade European III	CGAB	-	-	2711	France/Lorient 56	Litter	C. macrocarpa	PRJCA014243	SAMC1039041	CRX570960	C AA001708.1
BS255	A. bisporus var. bisporus	clade European III	CGAB	-	-	2702	France/St Malo 35	Litter	C. macrocarpa	PRJCA014243	SAMC1039042	CRX570961	C AA001746.1
BS175	A. bisporus var. bisporus	clade European III	CGAB	-	-	3165	France/Lorient 56	Litter	C. macrocarpa	PRJCA014243	SAMC1039043	CRX570962	C AA001707.1
BS367C	A. bisporus var. bisporus	clade European III	CGAB	-	-	2817	France/South-west 16	Manure	Goat or sheep	PRJCA014243	SAMC1039044	CRX570963	C AA001821.1
BS075D	A. bisporus var. bisporus	clade European III	CGAB	-	-	2755	France/Bordeaux 33	Litter	Picea	PRJCA014243	SAMC1039045	CRX570964	C AA001661.1
BS718	A. bisporus var. bisporus	clade European III	CGAB	-	-	2918	France/South-west 33	Litter	roadside embankments	PRJCA014243	SAMC1039046	CRX570965	C AA001967.1
BS102	A. bisporus var. bisporus	clade European III	ARP	FS-18	38	-	USA coastal Calif	Litter	C. macrocarpa	PRJCA014243	SAMC1039047	CRX570966	C AA001667.1
BS354B	A. bisporus var. bisporus	clade European III	CGAB	-	-	3241	France/ 49	Chicken manure	Goat or sheep or chickens)	PRJCA014243	SAMC1039048	CRX570967	C AA001811.1
BS159	A. bisporus var. bisporus	clade European III	ARP	FS-59	103	-	USA coastal Calif	Litter	C. macrocarpa	PRJCA014243	SAMC1039049	CRX570968	C AA001695.1
BS462	A. bisporus var. bisporus	clade European III	CGAB	-	-	2874	France/La Rochelle 17	Manure	Goat or sheep	PRJCA014243	SAMC1039050	CRX570969	C AA001856.1
BS583	A. bisporus var. bisporus	clade European III	CGAB	-	-	3332	France/South-west 33	Litter	white dune	PRJCA014243	SAMC1039051	CRX570970	C AA001887.1
BS012	A. bisporus var. bisporus	clade European III	PSUMCC	MC 351	-	2980	Canada-British Columbia	-	-	PRJCA014243	SAMC1039052	CRX570971	C AA001645.1
BS033	A. bisporus var. bisporus	clade European III	Somycel/Sylvan 56/456	-	-	2698	Cultivar brown	-	-	PRJCA014243	SAMC1039053	CRX570972	C AA001651.1
BS476	A. bisporus var. bisporus	clade European III	Diakov/ARP	43P	223	2891	Russia/Penza	-	-	PRJCA014243	SAMC1039054	CRX570973	C AA001859.1
BS287	A. bisporus var. bisporus	clade European III	CGAB	-	-	2806	France/South-west 33	Litter	C. macrocarpa	PRJCA014243	SAMC1039055	CRX570974	C AA001771.1
BS685	A. bisporus var. bisporus	clade European III	ARP	FS-70	260	-	USA Calofornia	-	-	PRJCA014243	SAMC1039056	CRX570975	C AA001949.1
BS146	A. bisporus var. bisporus	clade European III	ARP	FS-37	81	-	USA coastal Calif	Litter	C. macrocarpa; Goat or sheep	PRJCA014243	SAMC1039057	CRX570976	C AA001687.1
BS436A	A. bisporus var. bisporus	clade European III	CGAB	-	-	2821	France/La Rochelle 17	Litter	C. macrocarpa	PRJCA014243	SAMC1039058	CRX570977	C AA001847.1
BS589	A. bisporus var. bisporus	clade European III	ARP	AA0377	242	-	UK	-	-	PRJCA014243	SAMC1039059	CRX570978	C AA001891.1
BS434A	A. bisporus var. bisporus	clade European III	CGAB	-	-	3245	France/La Rochelle 17	Litter	C. macrocarpa	PRJCA014243	SAMC1039060	CRX570979	C AA001845.1
BS076C	A. bisporus var. bisporus	clade European III	CGAB	-	-	33336	France/Bordeaux 33	Litter	plant debris	PRJCA014243	SAMC1039061	CRX570980	C AA001662.1
BS439	A. bisporus var. bisporus	clade European III	ARP	SP-2	204	-	USA coastal Calif	Manure	compost	PRJCA014243	SAMC1039062	CRX570981	C AA001851.1
BS242	A. bisporus var. bisporus	clade European III	CGAB	-	-	2735	France/Lorient 56	Litter	C. macrocarpa	PRJCA014243	SAMC1039063	CRX570982	C AA001736.1
BS580	A. bisporus var. bisporus	clade European III	CGAB	-	-	2957	France/La Rochelle 17	Litter	C. macrocarpa	PRJCA014243	SAMC1039064	CRX570983	C AA001885.1
BS238	A. bisporus var. bisporus	clade European III	CGAB	-	-	2910	France/Lorient 56	Litter	C. macrocarpa	PRJCA014243	SAMC1039065	CRX570984	C AA001735.1
BS237	A. bisporus var. bisporus	clade European III	CGAB	-	-	2734	France/Lorient 56	Litter	C. macrocarpa	PRJCA014243	SAMC1039066	CRX570985	C AA001734.1
BS453	A. bisporus var. bisporus	clade European III	ARP	SP-1	203	-	USA coastal Calif	Manure	compost	PRJCA014243	SAMC1039067	CRX570986	C AA001854.1
BS702	A. bisporus var. bisporus	clade European III	CGAB	-	-	2839	France/South-west 33	Litter	under walnut tree plantation	PRJCA014243	SAMC1039068	CRX570987	C AA001959.1
BS435	A. bisporus var. bisporus	clade European III	CGAB	-	-	2834	France/La Rochelle 17	Litter	C. macrocarpa	PRJCA014243	SAMC1039069	CRX570988	C AA001846.1
BS438	A. bisporus var. bisporus	clade European III	CGAB	-	-	2819	France/La Rochelle 17	Litter	C. macrocarpa	PRJCA014243	SAMC1039070	CRX570989	C AA001850.1
BS437C	A. bisporus var. bisporus	clade European III	CGAB	-	-	2820	France/La Rochelle 17	Litter	C. macrocarpa	PRJCA014243	SAMC1039071	CRX570990	C AA001849.1
BS674	A. bisporus var. bisporus	clade European III	CGAB	-	-	3350	Greece/Thessaly	-	enclosure for sheep	PRJCA014243	SAMC1039072	CRX570991	C AA001945.1
BS618	A. bisporus var. bisporus	clade European III	CGAB	-	-	2949	Greece/Thessaly	Litter	Cupressus semp.	PRJCA014243	SAMC1039073	CRX570992	C AA001916.1
BS623	A. bisporus var. bisporus	clade European III	CGAB	-	-	3295	Greece/Thessaly	Litter	Cupressus semp.	PRJCA014243	SAMC1039074	CRX570993	C AA001920.1
BS147	A. bisporus var. bisporus	clade European III	ARP	FS-38	82	-	USA coastal Calif	Litter	C. macrocarpa	PRJCA014243	SAMC1039075	CRX570994	C AA001688.1
BS017	A. bisporus var. bisporus	clade European III	PSUMCC	AG 89	-	2981	Canada-Ontario	Litter	Bare ground	PRJCA014243	SAMC1039076	CRX570995	C AA001646.1
BS079B	A. bisporus var. bisporus	clade European III	CGAB	-	-	2775	France/ 53	Manure	Goat or sheep	PRJCA014243	SAMC1039077	CRX570996	C AA001665.1
BS003	A. bisporus var. bisporus	clade European III	Roy. C./France Mycelium 30A	-	-	3199	Cultivar small white	-	-	PRJCA014243	SAMC1039078	CRX570997	C AA001644.1
BS031	A. bisporus var. bisporus	clade European III	Somycel/Sylvan 65/765	-	-	3200	Cultivar small white	-	-	PRJCA014243	SAMC1039079	CRX570998	C AA001650.1
BS111	A. bisporus var. bisporus	clade European III	ARP	LM-1	31	-	Canada-Ontario	Litter	Poplars	PRJCA014243	SAMC1039080	CRX570999	C AA001673.1
BS494	A. bisporus var. bisporus	clade European III	CGAB	-	-	3261	Greece/Thessaly	Litter	Cupressus sempervirens	PRJCA014243	SAMC1039081	CRX571000	C AA001864.1
BS069A	A. bisporus var. bisporus	clade European III	CGAB	-	-	2958	France/ 91	Litter	Picea	PRJCA014243	SAMC1039082	CRX571001	C AA001655.1
BS069B	A. bisporus var. bisporus	clade European III	CGAB	-	-	3183	France/ 91	Litter	Picea	PRJCA014243	SAMC1039083	CRX571002	C AA001656.1
BS163	A. bisporus var. bisporus	clade European III	ARP	FS-63	107	-	USA coastal Calif	Litter	C. macrocarpa	PRJCA014243	SAMC1039084	CRX571003	C AA001697.1
BS172	A. bisporus var. bisporus	clade European III	CGAB	-	-	2688	France/Lorient 56	Litter	Pinus	PRJCA014243	SAMC1039085	CRX571004	C AA001705.1
BS85A-4	A. bisporus var. bisporus	clade European III	CGAB	-	-	2770	France/ 76	Litter	waste/plant debris	PRJCA014243	SAMC1039086	CRX571005	C AA001979.1
BS260	A. bisporus var. bisporus	clade European III	CGAB	-	-	2903	France/St Malo 35	Litter	C. macrocarpa	PRJCA014243	SAMC1039087	CRX571006	C AA001751.1
BS171	A. bisporus var. bisporus	clade European III	CGAB	-	-	2687	France/Lorient 56	Litter	C. macrocarpa	PRJCA014243	SAMC1039088	CRX571007	C AA001704.1
BS257	A. bisporus var. bisporus	clade European III	CGAB	-	-	2917	France/St Malo 35	Litter	C. macrocarpa	PRJCA014243	SAMC1039089	CRX571008	C AA001748.1
BS598	A. bisporus var. bisporus	clade European III	CGAB	-	-	2976	Greece/Island of Evia	Litter	Cupressus semp.	PRJCA014243	SAMC1039090	CRX571009	C AA001899.1
BS607	A. bisporus var. bisporus	clade European III	CGAB	-	-	2950	Greece/Thessaly	Litter	Cupressus semp.	PRJCA014243	SAMC1039091	CRX571010	C AA001908.1
BS660	A. bisporus var. bisporus	clade European III	CGAB	-	-	2971	Greece/Thessaly	Litter	Cupressus semp.	PRJCA014243	SAMC1039092	CRX571011	C AA001936.1
BS491	A. bisporus var. bisporus	clade European III	CGAB	-	-	2878	Greece/Thessaly	Litter	Cupressus sempervirens	PRJCA014243	SAMC1039093	CRX571012	C AA001861.1
BS493	A. bisporus var. bisporus	clade European III	CGAB	-	-	3260	Greece/Thessaly	Litter	Cupressus sempervirens	PRJCA014243	SAMC1039094	CRX571013	C AA001863.1
BS620	A. bisporus var. bisporus	clade European III	CGAB	-	-	2996	Greece/Thessaly	Litter	Cupressus semp.	PRJCA014243	SAMC1039095	CRX571014	C AA001917.1
BS611	A. bisporus var. bisporus	clade European III	CGAB	-	-	3328	Greece/Thessaly	Litter	Cupressus semp.+ Tuja	PRJCA014243	SAMC1039096	CRX571015	C AA001910.1
BS605	A. bisporus var. bisporus	clade European III	CGAB	-	-	2993	Greece/Thessaly	Litter	Cupressus semp.	PRJCA014243	SAMC1039097	CRX571016	C AA001906.1
BS610	A. bisporus var. bisporus	clade European III	CGAB	-	-	2951	Greece/Macedonia	Litter	Cupressus semp.	PRJCA014243	SAMC1039098	CRX571017	C AA001909.1
BS657	A. bisporus var. bisporus	clade European III	CGAB	-	-	2998	Greece/Thessaly	Litter	Cupressus semp.	PRJCA014243	SAMC1039099	CRX571018	C AA001935.1
BS604	A. bisporus var. bisporus	clade European III	CGAB	-	-	-	Greece/Thessaly	Litter	Cupressus semp.	PRJCA014243	SAMC1039100	CRX571019	C AA001905.1
BS664	A. bisporus var. bisporus	clade European III	CGAB	-	-	2999	Greece/Peloponese	Litter	Cupressus semp.	PRJCA014243	SAMC1039101	CRX571020	C AA001939.1
BS564	A. bisporus var. bisporus	clade European III	CGAB	-	-	3001	Greece/Island of Evia	Litter	Cupr. semp.	PRJCA014243	SAMC1039102	CRX571021	C AA001880.1
BS592	A. bisporus var. bisporus	clade European III	CGAB	-	-	2990	Greece/Thessaly	Litter	Cupressus semp.	PRJCA014243	SAMC1039103	CRX571022	C AA001893.1
BS621	A. bisporus var. bisporus	clade European III	CGAB	-	-	2940	Greece/Thessaly	Litter	Cupressus semp.	PRJCA014243	SAMC1039104	CRX571023	C AA001918.1
BS649	A. bisporus var. bisporus	clade European III	CGAB	-	-	3343	Greece/Thessaly	Litter	Cupressus semp.	PRJCA014243	SAMC1039105	CRX571024	C AA001930.1
BS600	A. bisporus var. bisporus	clade European III	CGAB	-	-	2992	Greece/Thessaly	Litter	Cupressus semp.	PRJCA014243	SAMC1039106	CRX571025	C AA001901.1
BS624	A. bisporus var. bisporus	clade European III	CGAB	-	-	2947	Greece/Thessaly	Litter	Cupressus semp.	PRJCA014243	SAMC1039107	CRX571026	C AA001921.1
BS614	A. bisporus var. bisporus	clade European III	CGAB	-	-	2948	Greece/Thessaly	Litter	Cupressus semp.	PRJCA014243	SAMC1039108	CRX571027	C AA001913.1
BS616	A. bisporus var. bisporus	clade European III	CGAB	-	-	2995	Greece/Thessaly	Litter	Cupressus semp.	PRJCA014243	SAMC1039109	CRX571028	C AA001914.1
BS613	A. bisporus var. bisporus	clade European III	CGAB	-	-	2994	Greece/Thessaly	Litter	Cupressus semp.	PRJCA014243	SAMC1039110	CRX571029	C AA001912.1
BS599	A. bisporus var. bisporus	clade European III	CGAB	-	-	2966	Greece/Thessaly	Litter	Cupressus semp.	PRJCA014243	SAMC1039111	CRX571030	C AA001900.1
BS113	A. bisporus var. bisporus	clade European III	ARP	FS-14	34	-	USA coastal Calif	Litter	C. macrocarpa	PRJCA014243	SAMC1039112	CRX571031	C AA001674.1
BS157	A. bisporus var. bisporus	clade European III	ARP	FS-55	99	-	USA coastal Calif	Litter	Juniperus	PRJCA014243	SAMC1039113	CRX571032	C AA001693.1
BS421B	A. bisporus var. bisporus	clade European III	CGAB	-	-	2901	France/South-west 40	Litter	C. macrocarpa	PRJCA014243	SAMC1039114	CRX571033	C AA001832.1
BS143	A. bisporus var. bisporus	clade European III	ARP	FS-33	77	-	USA coastal Calif	Litter	C. macrocarpa	PRJCA014243	SAMC1039115	CRX571034	C AA001685.1
BS367A	A. bisporus var. bisporus	clade European III	CGAB	-	-	3242	France/South-west 16	Sheep manure	Goat or sheep	PRJCA014243	SAMC1039116	CRX571035	C AA001820.1
BS036	A. bisporus var. bisporus	clade European III	Roy. C./France M. 202	-	-	3099	Cultivar small brown	-	-	PRJCA014243	SAMC1039117	CRX571036	C AA001653.1
BS184	A. bisporus var. bisporus	clade European III	CGAB	-	-	2716	France/Lorient 56	Litter	C. macrocarpa	PRJCA014243	SAMC1039118	CRX571037	C AA001714.1
BS690	A. bisporus var. bisporus	clade European III	CGAB	-	-	2838	France/Lorient 56	Litter	Tamarix in seaside	PRJCA014243	SAMC1039119	CRX571038	C AA001952.1
BS513	A. bisporus var. bisporus	clade European III	CGAB	-	-	3323	Greece/Thessaly	Manure	Goat or sheep	PRJCA014243	SAMC1039120	CRX571039	C AA001872.1
BS686	A. bisporus var. bisporus	clade European III	ARP	KP-2	261	-	USA-Massachussetts	-	-	PRJCA014243	SAMC1039121	CRX571040	C AA001950.1
BS200	A. bisporus var. bisporus	clade European III	CGAB	-	-	3168	France/Lorient 56	Litter	C. macrocarpa	PRJCA014243	SAMC1039122	CRX571041	C AA001717.1
BS512	A. bisporus var. bisporus	clade European III	CGAB	-	-	2777	Greece/Thessaly	Manure	Goat or sheep	PRJCA014243	SAMC1039123	CRX571042	C AA001871.1
BS640	A. bisporus var. bisporus	clade European III	CGAB	-	246	3341	France/Lorient 56	-	-	PRJCA014243	SAMC1039124	CRX571043	C AA001926.1
BS669	A. bisporus var. bisporus	clade European III	CGAB	-	-	2973	Greece/Macedonia	Litter	C. sempervirens	PRJCA014243	SAMC1039125	CRX571044	C AA001943.1
BS250	A. bisporus var. bisporus	clade European III	CGAB	-	-	2705	France/St Malo 22	Litter	C. macrocarpa	PRJCA014243	SAMC1039126	CRX571045	C AA001742.1
BS244	A. bisporus var. bisporus	clade European III	CGAB	-	-	2708	France/St Malo 22	Litter	C. macrocarpa	PRJCA014243	SAMC1039127	CRX571046	C AA001738.1
BS675	A. bisporus var. bisporus	clade European III	CGAB	-	-	3351	Greece/Thessaly	Manure	Goat or sheep	PRJCA014243	SAMC1039128	CRX571047	C AA001946.1
BS166	A. bisporus var. bisporus	clade European III	ARP	TV-2	112	-	USA-Wyoming	Litter	Dead Salix	PRJCA014243	SAMC1039129	CRX571048	C AA001700.1
BS502	A. bisporus var. bisporus	clade European III	CGAB	-	-	2889	Greece/Thessaly	Manure	Goat or sheep	PRJCA014243	SAMC1039130	CRX571049	C AA001866.1
BS697	A. bisporus var. bisporus	clade European III	CGAB	-	-	857	France/ 13	Litter	Abies, Picea	PRJCA014243	SAMC1039131	CRX571050	C AA001957.1
BS571	A. bisporus var. bisporus	clade European III	CGAB	-	-	3325	Greece/Thessaly	Manure	Goat or sheep	PRJCA014243	SAMC1039132	CRX571051	C AA001881.1
BS372	A. bisporus var. bisporus	clade European III	CGAB	-	-	3217	France/Bordeaux 33	Litter	C. arizonica	PRJCA014243	SAMC1039133	CRX571052	C AA001824.1
BS169	A. bisporus var. bisporus	clade European III	CGAB	-	-	2776	France/ 44	-	-	PRJCA014243	SAMC1039134	CRX571053	C AA001702.1
BS271	A. bisporus var. bisporus	clade European III	CGAB	-	-	2829	France/St Malo 35	Litter	C. macrocarpa	PRJCA014243	SAMC1039135	CRX571054	C AA001759.1
BS278	A. bisporus var. bisporus	clade European III	CGAB	-	-	2684	France/St Malo 35	Litter	C. macrocarpa	PRJCA014243	SAMC1039136	CRX571055	C AA001764.1
BS248	A. bisporus var. bisporus	clade European III	CGAB	-	-	2707	France/St Malo 22	Litter	C. macrocarpa	PRJCA014243	SAMC1039137	CRX571056	C AA001740.1
BS256	A. bisporus var. bisporus	clade European III	CGAB	-	-	2827	France/St Malo 35	Litter	C. macrocarpa	PRJCA014243	SAMC1039138	CRX571057	C AA001747.1
BS691	A. bisporus var. bisporus	clade European III	CGAB	-	-	2825	France/Lorient 56	Litter	cypress and cut grass	PRJCA014243	SAMC1039139	CRX571058	C AA001953.1
BS251	A. bisporus var. bisporus	clade European III	CGAB	-	-	2706	France/St Malo 22	Litter	C. macrocarpa	PRJCA014243	SAMC1039140	CRX571059	C AA001743.1
BS277	A. bisporus var. bisporus	clade European III	CGAB	-	-	3158	France/St Malo 35	Litter	C. macrocarpa	PRJCA014243	SAMC1039141	CRX571060	C AA001763.1

Supplementary Table S2 IDP for each strain

Strain	Clade	Intron Distribution Pattern (number of introns in cox1)	wild, cultivar or cultivar-like	location	nad5 intron1 Group IB	nad5 intron2 Group ID	nad5 intron3 Group IC2	nad5 intron4 Group IB	nad5 intron5 Group IB	nad5 intron6 Group IB	nad5 intron7 Group II
A.sinodeliciosus	A. sinodeliciosus	(20)	wild	China Xinjiang	present	present	present	present	present	present	present
ARP276	A. cf. subfloccosus	(15)	wild	USA New mexico	present	present	present	present	absent	absent	present
QL20170217	A. qilianensis	(19)	wild	China qilian mountain	present	present	present	present	absent	absent	present
ZRL20161973		(20)	wild	China qilian mountain	present	present	present	present	absent	absent	present
QL20162067		(19)	wild	China qilian mountain	present	present	present	present	absent	absent	present
QL20170258	A. sinotetrasporus	(20)	wild	China qilian mountain	present	present	present	present	present	absent	present
ZRL20161862		(20)	wild	China qilian mountain	present	present	present	present	present	absent	present
QL20180590			wild	China qilian mountain	present	present	present	present	present	absent	present
ZRL20161020			wild	China qilian mountain	present	present	present	present	present	absent	present
BS423	bisporus eurotetrasporus	IDP1 (22)	wild	France/La Rochelle 85	present	present	absent	present	absent	absent	present
BS514			wild	Greece/Thessaly	present	present	absent	present	absent	absent	present
BS321-11	bisporus Europe II	IDP2 (20)	wild	USA Californian Desert	present	present	absent	present	absent	absent	present
BS321-21			wild	USA Californian Desert	present	present	absent	present	absent	absent	present
BS059		IDP3 (20)	cultivar white	Le Lion B92	present	present	absent	present	absent	absent	present
BS034			cultivar white	Somycel 53	present	present	absent	present	absent	absent	present
BS021			cultivar white	Somycel 91	present	present	absent	present	absent	absent	present
BS026-7			cultivar hybrid U1	hybrid cultivar	present	present	absent	present	absent	absent	present
U1			cultivar hybrid U1	cultivated in China	present	present	absent	present	absent	absent	present
BS403			cultivar hybrid	hybrid cultivar	present	present	absent	present	absent	absent	present
3X			cultivar	cultivated in China	present	present	absent	present	absent	absent	present
As2796			cultivar	cultivated in China	present	present	absent	present	absent	absent	present
W192			cultivar	cultivated in China	present	present	absent	present	absent	absent	present
901			cultivar	cultivated in China	present	present	absent	present	absent	absent	present
W38			cultivar	cultivated in China	present	present	absent	present	absent	absent	present
A15			cultivar	cultivated in China	present	present	absent	present	absent	absent	present
W2000			cultivar	cultivated in China	present	present	absent	present	absent	absent	present
[illegible]	[illegible]	[illegible]	[illegible]	[illegible]	[illegible]	[illegible]	[illegible]	[illegible]	[illegible]	[illegible]	[illegible]

Strain	cob intron1 Group IB	cob intron2 Group IB	cob intron3 Group IB	cob intron4 Group IC2	cob intron5 Group ID	cob intron6 Group ID	cob intron7 Group IC2	cob intron8 Group ID	cob intron9 Group IA	cob intron10 Group IB	cob intron11 Group IB	cob intron12 Group IB	cob intron13 Group IB
A.sinodeliciosus	present	present	present	present	present	present	present	present	present	present	present	present	present
ARP276	present	present	present	present	present	present	absent	present	absent	absent	absent	absent	present
QL20170217	present	present	present	present	present	present	absent	present	absent	absent	present	absent	present
ZRL20161973	present	present	present	present	present	present	absent	present	absent	absent	present	absent	present
QL20162067	present	present	present	present	present	present	absent	present	absent	absent	present	absent	present
QL20170258	present	present	present	present	present	present	absent	present	absent	absent	present	absent	present
ZRL20161862	present	present	present	present	present	present	absent	present	absent	absent	present	absent	present
QL20180590	present	present	present	present	present	present	absent	present	absent	absent	present	absent	present
ZRL20161020	present	present	present	present	present	present	absent	present	absent	absent	present	absent	present
BS423	present	present	present	present	present	present	absent	present	absent	absent	present	absent	absent
BS514	present	present	present	present	present	present	absent	present	absent	absent	present	absent	absent
BS321-11	present	present	present	present	present	present	absent	present	absent	absent	present	absent	absent
BS321-21	present	present	present	present	present	present	absent	present	absent	absent	present	absent	absent
BS059	present	present	present	present	present	present	absent	present	absent	absent	present	absent	present
BS034	present	present	present	present	present	present	absent	present	absent	absent	present	absent	present
BS021	present	present	present	present	present	present	absent	present	absent	absent	present	absent	present
BS026-7	present	present	present	present	present	present	absent	present	absent	absent	present	absent	present
U1	present	present	present	present	present	present	absent	present	absent	absent	present	absent	present
BS403	present	present	present	present	present	present	absent	present	absent	absent	present	absent	present
3X	present	present	present	present	present	present	absent	present	absent	absent	present	absent	present
As2796	present	present	present	present	present	present	absent	present	absent	absent	present	absent	present
W192	present	present	present	present	present	present	absent	present	absent	absent	present	absent	present
901	present	present	present	present	present	present	absent	present	absent	absent	present	absent	present
W38	present	present	present	present	present	present	absent	present	absent	absent	present	absent	present
A15	present	present	present	present	present	present	absent	present	absent	absent	present	absent	present
W2000	present	present	present	present	present	present	absent	present	absent	absent	present	absent	present
[illegible]	[illegible]	[illegible]	[illegible]	[illegible]	[illegible]	[illegible]	[illegible]	[illegible]	[illegible]	[illegible]	[illegible]	[illegible]	[illegible]

Strain	cox2 intron1 Group I	cox2 intron2 Group IB	cox2 intron3 Group ID	cox2 intron4 Group IC2	cox2 intron5 Group IB	cox2 intron6 Group IC1	cox1 intron1 Group II	cox1 intron2 Group II	cox1 intron3 Group IC2	cox1 intron4 Group II	cox1 intron5 Group IB	cox1 intron6 Group IB	cox1 intron7 Group IB(5')	cox1 intron8 Group IB	cox1 intron9 Group IB(3')	cox1 intron10 Group IB	cox1 intron11 Group IB	cox1 intron12 up ID 2/Gr	cox1 intron13 Group IB
A.sinodeliciosus	present	absent	absent	present	present	present	present	absent	absent	absent	present	present	present	present	absent	present	absent	type2	present
ARP276	present	present	present	absent	present	present	absent	present	part	part	absent	absent	present	absent	absent	present	absent	type1	present
QL20170217	present	present	present	absent	present	present	absent	absent	absent	absent	insert(19bp	present	present	present	absent	present	absent	type1	present
ZRL20161973	present	present	present	absent	present	present	absent	absent	absent	absent	insert(19bp	present	present	present	absent	present	present	type1	present
QL20162067	present	present	present	absent	present	present	absent	absent	absent	absent	insert(19bp	present	present	present	absent	present	absent	type1	present
QL20170258	present	present	present	absent	present	present	absent	absent	absent	absent	insert(19bp	present	present	absent	present	present	present	type1	present
ZRL20161862	present	present	present	absent	present	present	absent	absent	absent	absent	insert(19bp	present	present	absent	present	present	present	type1	present
QL20180590	present	present	present	absent	present	present	absent	absent	absent	absent	insert(19bp	present	present	absent	present	present	present	type1	present
ZRL20161020	present	present	present	absent	present	present	absent	absent	absent	absent	insert(19bp	present	present	absent	present	present	present	type1	present
BS423	present	absent	absent	absent	absent	present	absent	absent	present	present	present	present	present	present	present	present	present	type2	insert(1238
BS514	present	absent	absent	absent	absent	present	absent	absent	present	present	present	present	present	present	present	present	present	type2	insert(1238
BS321-11	present	absent	absent	absent	absent	present	present	absent	present	present	present	present	present	present	present	present	present	type2	present
BS321-21	present	absent	absent	absent	absent	present	present	absent	present	present	present	present	present	present	present	present	present	type2	present
BS059	present	absent	absent	absent	absent	present	absent	absent	present	present	present	present	present	present	present	present	present	type2	present
BS034	present	absent	absent	absent	absent	present	absent	absent	present	present	present	present	present	present	present	present	present	type2	present
BS021	present	absent	absent	absent	absent	present	absent	absent	present	present	present	present	present	present	present	present	present	type2	present
BS026-7	present	absent	absent	absent	absent	present	absent	absent	present	present	present	present	present	present	present	present	present	type2	present
U1	present	absent	absent	absent	absent	present	absent	absent	present	present	present	present	present	present	present	present	present	type2	present
BS403	present	absent	absent	absent	absent	present	absent	absent	present	present	present	present	present	present	present	present	present	type2	present
3X	present	absent	absent	absent	absent	present	absent	absent	present	present	present	present	present	present	present	present	present	type2	present
As2796	present	absent	absent	absent	absent	present	absent	absent	present	present	present	present	present	present	present	present	present	type2	present
W192	present	absent	absent	absent	absent	present	absent	absent	present	present	present	present	present	present	present	present	present	type2	present
901	present	absent	absent	absent	absent	present	absent	absent	present	present	present	present	present	present	present	present	present	type2	present
W38	present	absent	absent	absent	absent	present	absent	absent	present	present	present	present	present	present	present	present	present	type2	present
A15	present	absent	absent	absent	absent	present	absent	absent	present	present	present	present	present	present	present	present	present	type2	present
W2000	present	absent	absent	absent	absent	present	absent	absent	present	present	present	present	present	present	present	present	present	type2	present
[illegible]	[illegible]	[illegible]	[illegible]	[illegible]	[illegible]	[illegible]	[illegible]	[illegible]	[illegible]	[illegible]	[illegible]	[illegible]	[illegible]	[illegible]	[illegible]	[illegible]	[illegible]	[illegible]	[illegible]

Strain	cox1 intron14 Group IB	cox1 intron15 up IB 1/Gr	cox1 intron16 Group ID	cox1 intron17 Group IB	cox1 intron18 Group IB	cox1 intron19 Group IB	cox1 intron20 Group IB	cox1 intron21 Group IB	cox1 intron22 Group IB	cox1 intron23 /Group I(de	cox1 intron24 Group IB	cox1 intron25 Group IB	cox1 intron26 1/Group IA 2/G	nad4 intron1 Group II	nad4 intron2 Group IC2	nad4 intron3 Group IB	nad1 intron1 Group IC2	nad1 intron2 Group IB	nad1 intron3 Group IB	cox3 intron1 Group IB	cox3 intron2 Group ID
A.sinodeliciosus	present	type2	present	present	present	present	present	present	present	type3	present	absent	type1	present	present	present	present	present	present	present	present
ARP276	absent	absent	present	present	insert(1162	present	absent	present	present	type3	absent	absent	type1	absent	present	present	present	present	present	absent	absent
QL20170217	present	type2	present	present	insert(1162	present	present	present	present	type1	present	absent	type1	present	present	present	absent	present	present	present	absent
ZRL20161973	present	type2	present	present	insert(1162	present	present	present	present	type2	present	absent	type1	present	present	present	absent	present	present	present	absent
QL20162067	present	type2	present	present	present	present	present	present	present	type2	present	absent	type1	present	present	present	absent	present	present	present	absent
QL20170258	present	type2	present	present	insert(1162	present	present	present	present	type2	present	absent	type1	absent	absent	absent	absent	present	present	present	absent
ZRL20161862	present	type2	present	present	insert(1162	present	present	present	present	type2	present	absent	type1	present	present	absent	absent	present	present	present	absent
QL20180590	present	type2	present	present	insert(1162	present	present	present	present	type2	present	absent	type1	present	present	absent	absent	present	present	present	absent
ZRL20161020	present	type2	present	present	insert(1162	present	present	present	present	type2	present	absent	type1	present	present	absent	absent	present	present	present	absent
BS423	absent	absent	present	present	present	present	present	present	present	type2	present	present	type2	absent	absent	present	absent	present	present	present	absent
BS514	absent	absent	present	present	present	present	present	present	present	type2	present	present	type2	absent	absent	present	absent	present	present	present	absent
BS321-11	absent	absent	present	present	present	absent	present	present	present	type1	absent	absent	type2	absent	absent	present	absent	present	present	present	absent
BS321-21	absent	absent	present	present	present	absent	present	present	present	type1	absent	absent	type2	absent	absent	present	absent	present	present	present	absent
BS059	absent	absent	present	present	present	absent	present	present	present	type3	present	absent	type2	absent	absent	present	absent	present	present	present	absent
BS034	absent	absent	present	present	present	absent	present	present	present	type3	present	absent	type2	absent	absent	present	absent	present	present	present	absent
BS021	absent	absent	present	present	present	absent	present	present	present	type3	present	absent	type2	absent	absent	present	absent	present	present	present	absent
BS026-7	absent	absent	present	present	present	absent	present	present	present	type3	present	absent	type2	absent	absent	present	absent	present	present	present	absent
U1	absent	absent	present	present	present	absent	present	present	present	type3	present	absent	type2	absent	absent	present	absent	present	present	present	absent
BS403	absent	absent	present	present	present	absent	present	present	present	type3	present	absent	type2	absent	absent	present	absent	present	present	present	absent
3X	absent	absent	present	present	present	absent	present	present	present	type3	present	absent	type2	absent	absent	present	absent	present	present	present	absent
As2796	absent	absent	present	present	present	absent	present	present	present	type3	present	absent	type2	absent	absent	present	absent	present	present	present	absent
W192	absent	absent	present	present	present	absent	present	present	present	type3	present	absent	type2	absent	absent	present	absent	present	present	present	absent
901	absent	absent	present	present	present	absent	present	present	present	type3	present	absent	type2	absent	absent	present	absent	present	present	present	absent
W38	absent	absent	present	present	present	absent	present	present	present	type3	present	absent	type2	absent	absent	present	absent	present	present	present	absent
A15	absent	absent	present	present	present	absent	present	present	present	type3	present	absent	type2	absent	absent	present	absent	present	present	present	absent
W2000	absent	absent	present	present	present	absent	present	present	present	type3	present	absent	type2	absent	absent	present	absent	present	present	present	absent
[illegible]	[illegible]	[illegible]	[illegible]	[illegible]	[illegible]	[illegible]	[illegible]	[illegible]	[illegible]	[illegible]	[illegible]	[illegible]	[illegible]	[illegible]	[illegible]	[illegible]	[illegible]	[illegible]	[illegible]	[illegible]	[illegible]

Figure Legends:

Supplementary Figure 1. Mitogenome annotation for each strain.

This diagram shows the mitogenome annotation information for each strain, arranged in the order of the phylogenomic tree. Different types of genes are represented using different shapes.

Supplementary Figure 2. Positions and distributions of SNPs in the reference genome.

The outermost ring shows the reference genome JX27275 and its annotation information, with exons indicated in orange and introns in blue. The inner three rings indicate the location of SNPs, with the innermost ring marking all filtered SNP sites (dataset1), the middle ring indicating SNP sites on introns and the outer ring indicating SNP sites on exons(dataset2). Where dataset1 and dataset2 are used for phylogenetic analysis.

Supplementary Figure 3. Phylogenomic and population structure analysis.

1666 SNP data from the mitochondrial genomes of 361 strains were used. The Outgroup and the seven clades in which of *A. bisporus* are divided are marked in the figure. The genuine wild strains, cultivar-like strains and cultivar strains are distinguished by a different color on the strain name. The outer circles show the results of the population structure analysis for K values of 3 to 9, respectively.

Supplementary Figure 4. CV error of each K values in population structure analysis.

K values are set from 2 to 20 and the CV error value for each K value is shown in the diagram.

Supplementary Figure 5. Three evolutionary scenarios compared.

To simplify the topology, Clade Europe I and II, Clade America I and II were merge. The five clades make up a total of three possible scenario (topology), representing the different population historiy of *A. bisporus.*

Supplementary Figure 6. Projection of three scenarios on the first two LDA axes.

The highest RF classification votes and posterior probabilities were for scenario 1.

Supplementary Figure 7. Different intron types at the same site within the *cox1* gene.

(A). Intron 3 and 4. Intron degeneration was found in *A.* cf. *subfloccosus*, where partial sequences of intron 3 and intron 4 were deleted. (B). Intron 12. In this site type 1 and type 2 are two different sequences and there are differences in end of exon 12. Type 1 belongs to group ID presented in *A. subfloccosus*, *A. qilianensis*, *A. sinotetrasporus*, Clades America II, Europe II, III of *A. bisporus*, which has been reported (Férandon et al. 2010; 2013); Type 2 belongs to group IB presented in *A. sinodeliciosus* and all *A. bisporus* clade which has been named iAbi9' by Banafsheh (Banafsheh et al. 2015). (C). Intron13. In this site, some strains had an intron inserted by a 1238 bp sequence with a mutation near the insertion site. The insertion sequence has a high similarity to the cox1 intron of *Trametes cingulate* (69.33% identities). (D). Intron 15. In this site type 1 and type 2 are two different sequences and there are differences in end of exon 15 and the start of exon16. Type 1 belongs to group IB and present in Clades Europe II, III had been reported in JX271275 (Férandon et al. 2013); Type 2 belongs to group ID present in *A. sinodeliciosus*, *A. qilianensis*, and *A. sinotetrasporus* which has 84% similarity to the cox1 intron sequence of *Coprinus comatus*. (E). Intron 18. In this site, *A.* cf. *subfloccosus*, *A. qilianensis*, and *A. sinotetrasporus* had an intron inserted by a 1158 bp sequence with a mutation near the insertion site. The insertion sequence has a high similarity to the cox1 intron of *Tuber calosporum* (73% of identities) and *Dactylella tenuis* (78% of identities). (F). Intron 23. In this site type 1, type 2, and type 3 are three different sequences and there are differences in start of exon 24. Type1 belongs to group I presented in *A. qilianensis* and Clade America II which has 77% similarity to the cox1 intron sequence of *Endoconidiophora resinifera*; and Type2 has 74% similarity to the cox1 intron sequence of *Trametes hirsute*; Type3 belongs to group IB presented in the *A. sinodeliciosus*, *A. subfloccosus*, Clades China, and Europe I, II, III had been reported in JX271275 (Férandon et al. 2013). (G). Intron 26. In this site type 1 and type 2 are two different sequences and no mutations in the exons on either side. Type1 belongs to group IA present in the four outgroup species which

has 86% similarity to the cox1 intron sequence of *Ganoderma subamboinense*; Type2 present in all *A. bisporus* samples.

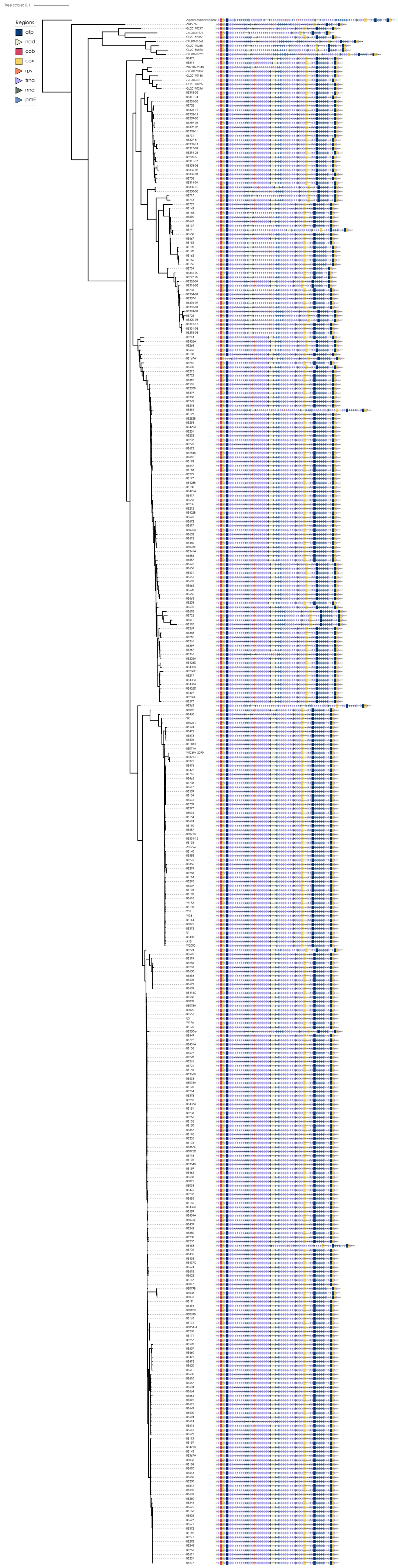

Supplementary Figure 1

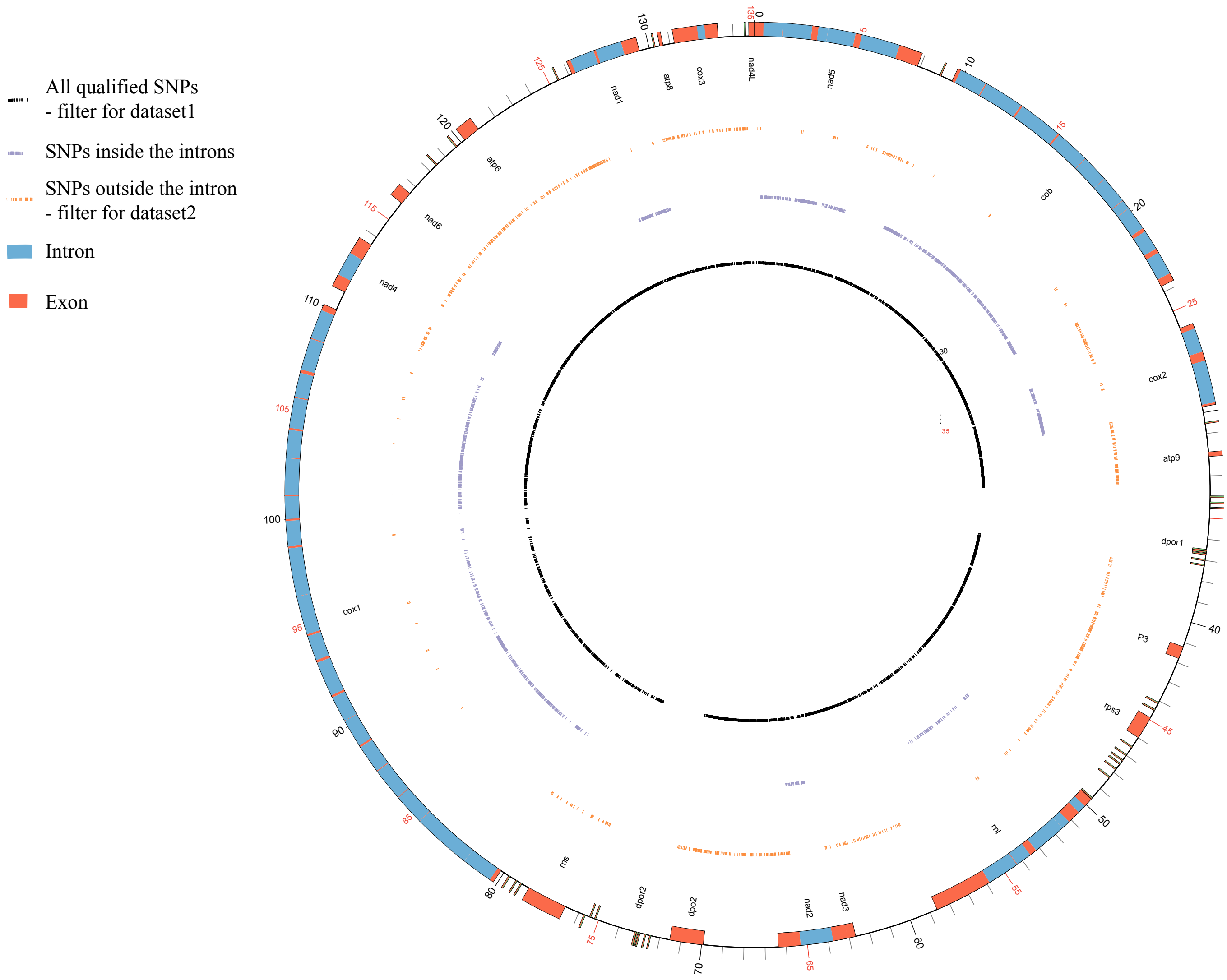

Supplementary Figure 2

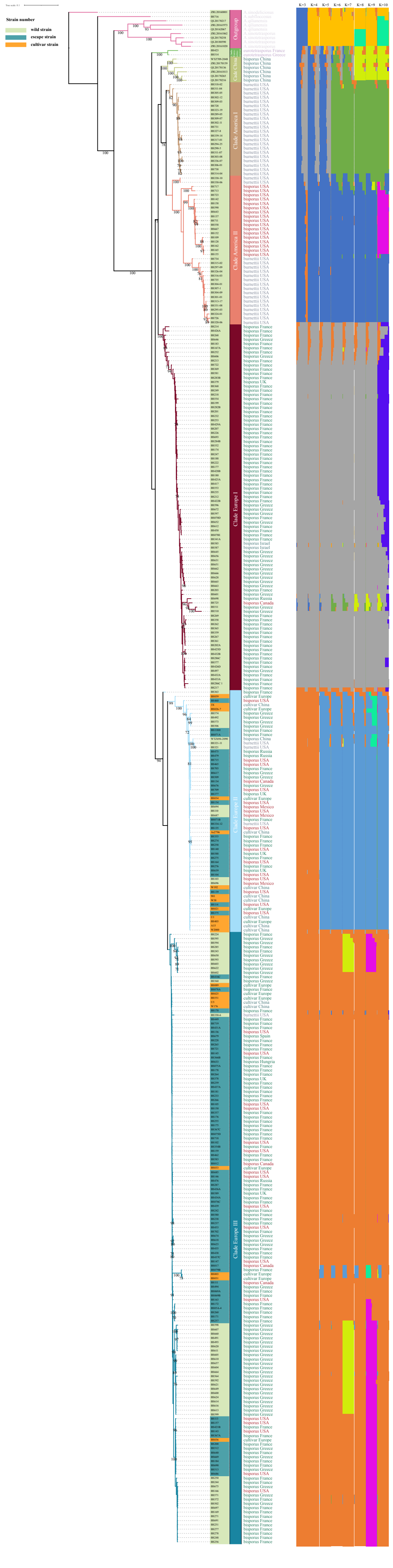

Supplementary Figure 3

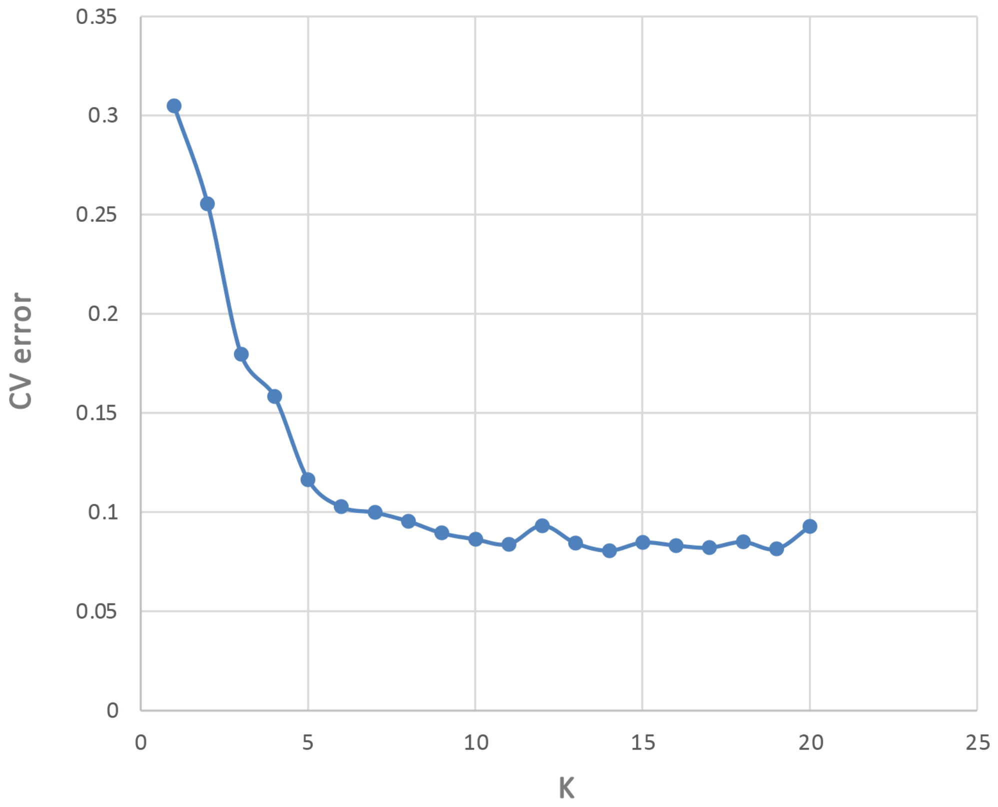

Supplementary Figure 4

t4

t3

t2

t1

0

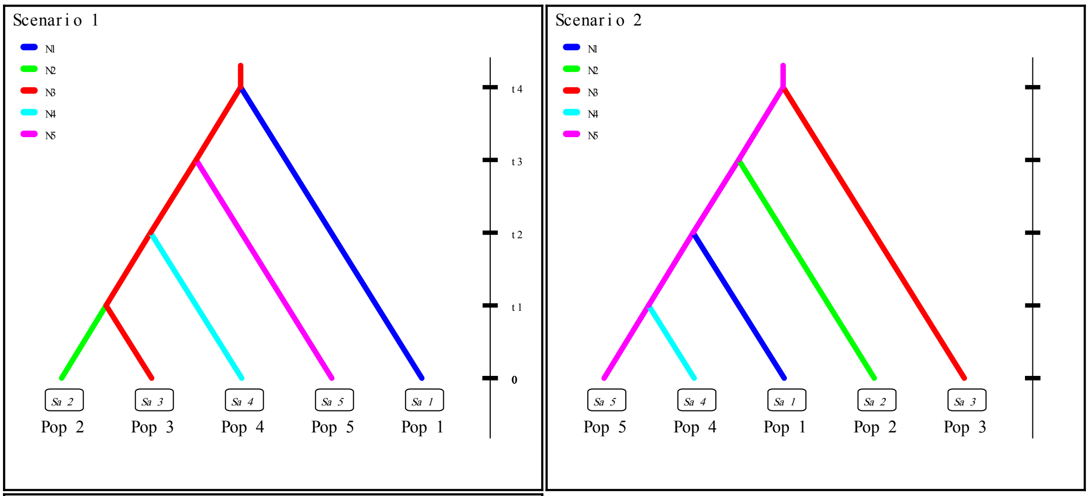

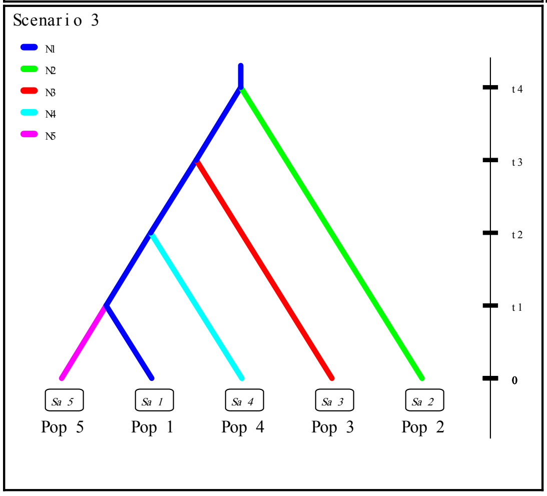

Pop1 Clade Eurotetrasporus

Pop2 Clade China

Pop3 Clade America I + II

Pop4 Clade Europe I

Pop5 Clade Europe II + III

Supplementary Figure 5

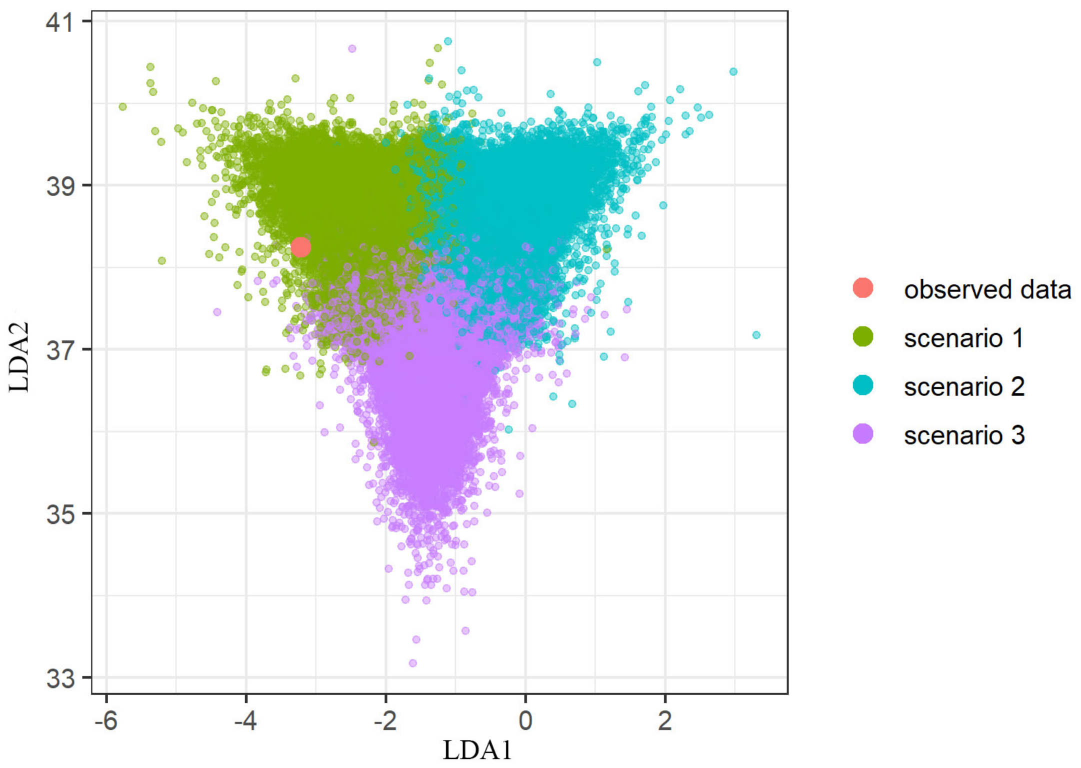

Supplementary Figure 6

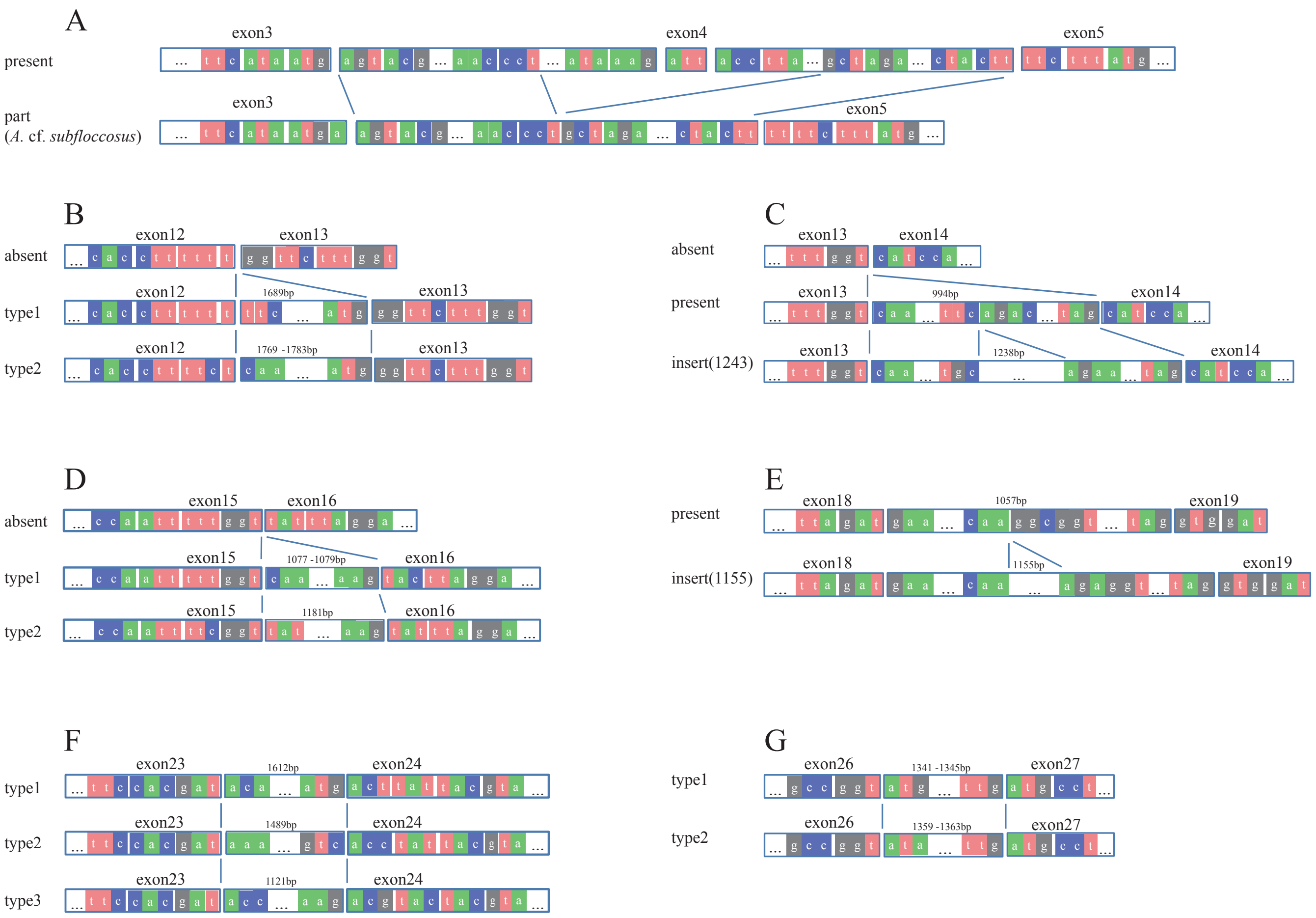

Supplementary Figure 7
